# Two essential Thioredoxins mediate apicoplast biogenesis, protein import, and gene expression in *Toxoplasma gondii*

**DOI:** 10.1371/journal.ppat.1006836

**Published:** 2018-02-22

**Authors:** Marco Biddau, Anne Bouchut, Jack Major, Tracy Saveria, Julie Tottey, Ojore Oka, Marcel van-Lith, Katherine Elizabeth Jennings, Jana Ovciarikova, Amy DeRocher, Boris Striepen, Ross Frederick Waller, Marilyn Parsons, Lilach Sheiner

**Affiliations:** 1 Wellcome Centre for Molecular Parasitology, University of Glasgow, 120 University Place Glasgow, United Kingdom; 2 Center for Infectious Disease Research, Seattle, WA, United States of America; 3 Institute of Molecular Cell and Systems Biology, Wolfson Link Building, University of Glasgow, Glasgow, United Kingdom; 4 Center for Tropical & Emerging Global Diseases, University of Georgia, Brooks Dr. Athens, GA, United States of America; 5 Department of Biochemistry, University of Cambridge, Cambridge, United Kingdom; 6 Department of Global Health, University of Washington, Seattle, WA, United States of America; Francis Crick Institute, UNITED KINGDOM

## Abstract

Apicomplexan parasites are global killers, being the causative agents of diseases like toxoplasmosis and malaria. These parasites are known to be hypersensitive to redox imbalance, yet little is understood about the cellular roles of their various redox regulators. The apicoplast, an essential plastid organelle, is a verified apicomplexan drug target. Nuclear-encoded apicoplast proteins traffic through the ER and multiple apicoplast sub-compartments to their place of function. We propose that thioredoxins contribute to the control of protein trafficking and of protein function within these apicoplast compartments. We studied the role of two *Toxoplasma gondii* apicoplast thioredoxins (*Tg*ATrx), both essential for parasite survival. By describing the cellular phenotypes of the conditional depletion of either of these redox regulated enzymes we show that each of them contributes to a different apicoplast biogenesis pathway. We provide evidence for *Tg*ATrx1’s involvement in ER to apicoplast trafficking and *Tg*ATrx2 in the control of apicoplast gene expression components. Substrate pull-down further recognizes gene expression factors that interact with *Tg*ATrx2. We use genetic complementation to demonstrate that the function of both *Tg*ATrxs is dependent on their disulphide exchange activity. Finally, *Tg*ATrx2 is divergent from human thioredoxins. We demonstrate its activity *in vitro* thus providing scope for drug screening. Our study represents the first functional characterization of thioredoxins in *Toxoplasma*, highlights the importance of redox regulation of apicoplast functions and provides new tools to study redox biology in these parasites.

## Author summary

To survive, apicomplexan parasites must adjust to the redox insults they experience. These parasites undergo redox stresses induced by the host cell within which they live, by the host immune system, and by their own metabolic activities. Yet the myriad of cellular processes that are affected by redox changes and that may take part in maintaining the redox balance within the parasite are largely understudied. Thioredoxins are enzymes that link the redox state of subcellular environments to the functional state or the cellular trafficking of their substrate proteins. In this work, we identify two pathways that are controlled by two thioredoxins in the apicomplexan *Toxoplasma gondii*, and demonstrate that both are essential for parasite survival. We show that each of these enzymes contributes to the function of the apicomplexan plastid, the apicoplast, a unique parasite organelle with importance for drug discovery efforts. We thus highlight that part of the apicomplexan sensitivity to redox imbalance is specifically related to the apicoplast, and point at the importance of thioredoxins in mediating apicoplast biogenesis. Finally, our work raises the potential of apicoplast thioredoxins as new drug targets.

## Introduction

Apicomplexan parasites are global killers of animals and humans. Most apicomplexans possess a plastid, the apicoplast. The apicoplast is essential for parasite survival [1] throughout their complex life cycles and has no equivalent in humans. Accordingly, apicoplast functions and pathways of biogenesis are sought after as promising drug targets for diseases like toxoplasmosis and malaria [2].

**Fig 1:**
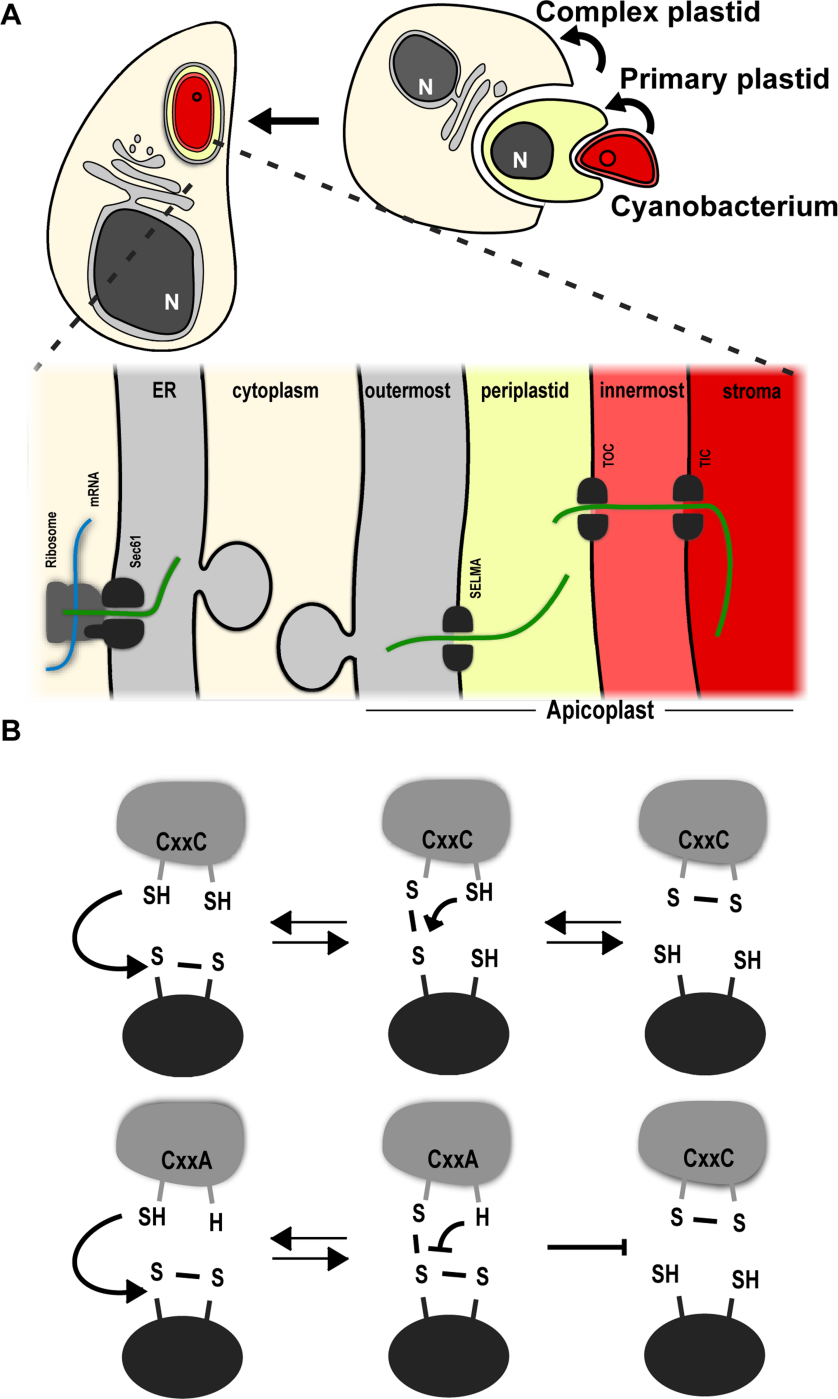
Illustrations of the two main processes studied in this work. **(A)** Complex plastid evolution and the resulting architecture and protein trafficking starting from the co-translational (mRNA in blue, new polypeptide in green) translocation into the ER (light grey) through the four apicoplast compartments (grey, yellow, light-red, red) via their corresponding translocons (depicted as dark grey split ovals): Sec61, SELMA (symbiont-specific ERAD (endoplasmic reticulum-associated degradation)-like machinery), TOC/TIC (translocon of the outer/inner chloroplast membrane) **(B)** Disulphide exchange between Trx (light-grey) and its substrate (dark-grey oval), and of its dependence on the CXXC motif.

The apicoplast was acquired via endosymbiosis, whereby a eukaryotic auxotroph took up an autotrophic alga (Fig 1A). Subsequent reduction of the algal organelles and gene transfer from the algal nuclear genome to the auxotroph genome resulted in integration of the alga as an organelle. A similar pathway gave rise also to the complex plastids found in a divergent group of organisms of ecological or medical importance. These include the cryptophytes, heterokonts, haptophytes, dinoflagellates, and chromerids [3]. The apicoplast and related plastids have multiple compartments: the two inner compartments originate from the algal primary plastid. The next compartment out is the periplastid compartment (PPC). The PPC is remnant of the algal cytosol, and in the cryptophyte plastid the PPC still hosts a relic of the algal nucleus, named the nucleomorph [4]. Finally, the outermost compartment comes from the host and is either of phagosomal [5] or ER [6] origin. Most apicoplast proteins are nuclear encoded, co-translationally translocated into the ER and trafficked from there to the apicoplast outermost membrane and through the subsequent compartments to their destination within the organelle.

The conservation of this elaborate architecture necessitates a mechanism that ensures that proteins fold into their functional forms in their appropriate compartment and that they are kept in a conformation compatible with their translocation through the membranes bounding these compartments. The formation of disulphide bonds from dithiols affects the conformation of proteins and thus plays an important regulatory role in their sorting to their target cellular compartments and in their correct function when in these compartments. Proteins with thioredoxin domains (Trxs) mediate disulphide-dithiol dynamics in target proteins in response to compartmental redox states. The thioredoxin (Trx) fold contains a double cysteine active site (cysteine-X-X-cysteine/CXXC) through which the disulphide exchange occurs. In this reaction, the N-terminal cysteine serves as a nucleophile, creating an intermediate mixed disulphide species with the substrate. Next, a nucleophilic attack by the C-terminal cysteine of the Trx CXXC motif on its N-terminal cysteine results in an oxidized CXXC motif and in the release of the reduced substrate (Fig 1B illustrates this process). A well-studied example of the role of Trxs in controlling protein folding and sorting is the family of protein disulphide isomerases (PDI) that mediate the folding and sorting of secretory proteins in the ER [7]. Redox mediated protein folding also takes place in the mitochondrial intermembrane space via the oxidoreductase Mia40 and the oxidase Erv1 [8]. We have previously identified two apicoplast Trxs in *Toxoplasma gondii* (*Tg*ATrxs). *Tg*ATrx1 is a resident of the apicoplast periphery and is also found in vesicles in the cytosol, around the apicoplast and at low levels in the ER [9]. *Tg*ATrx2, also found at the apicoplast periphery, was suggested to be a PPC resident, which is compatible with the presence of its orthologues in the cryptophyte nucleomorph genomes [10]. The roles of both *Tg*ATrxs are unknown. Here we examine the hypothesis that *Tg*ATrxs may play a role in the control of apicoplast biogenesis by mediating disulphide exchange with proteins destined to different compartments of the apicoplast. We show that both *Tg*ATrxs are essential for parasite growth. In line with our hypothesis both *Tg*ATrxs depend on their CXXC active site for function, but the depletion of each *Tg*ATrx results in a defect in a different apicoplast biogenesis pathway. Finally, *Tg*ATrx2 and its malaria orthologues have features that are divergent from canonical Trxs. Nevertheless, recombinant *Tg*ATrx2 exchanges disulphide *in vitro*. We have utilized this ability to generate an *in vitro* activity assay that lays the foundations for the development of a platform for drug screens in the future.

## Results

### The two ATrxs have distinct evolutionary origins and their staining patterns suggest differences in their sub-compartmental localizations

**Fig 2:**
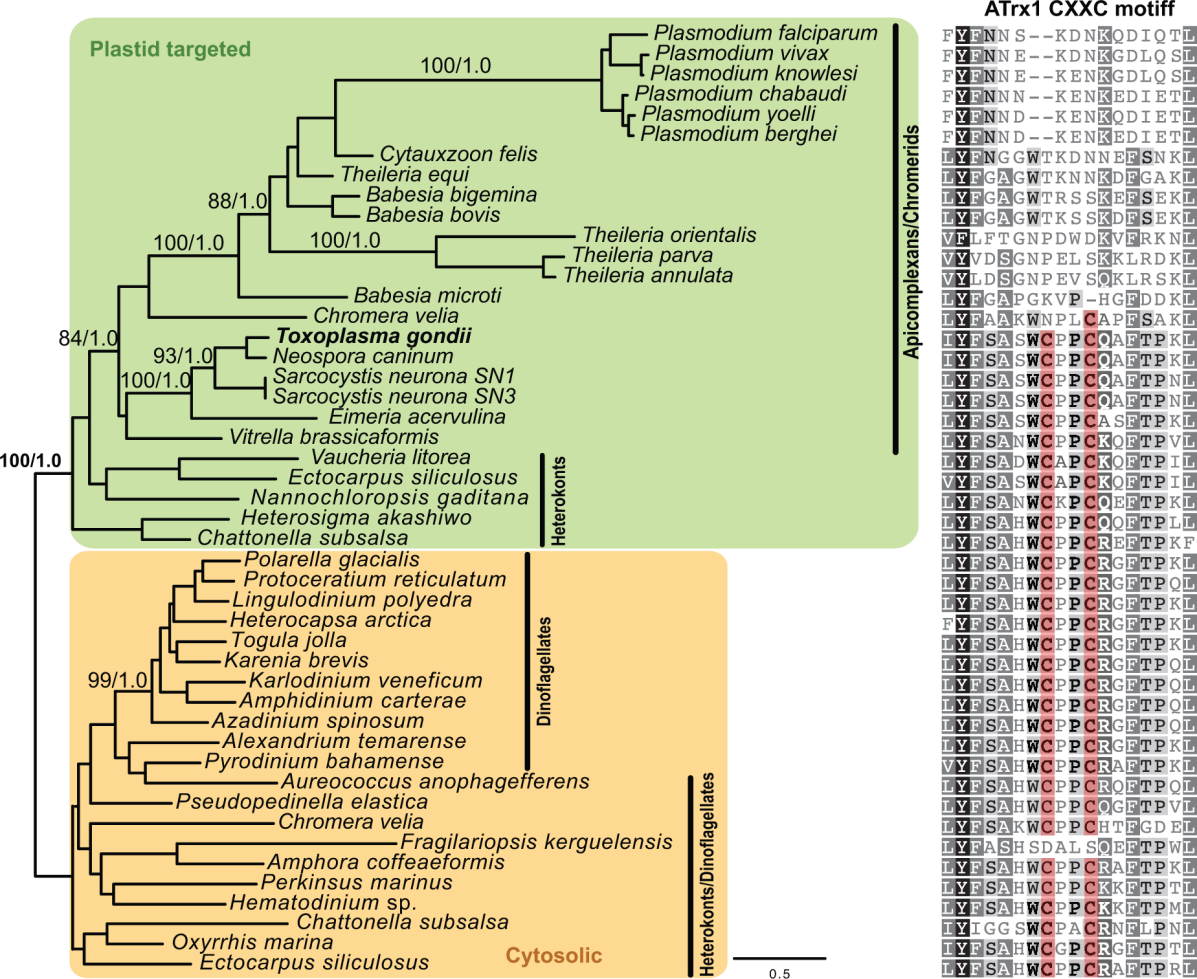
Phylogenetic analysis of ATrx1 sequences. ATrx1 phylogeny and conservation of the CXXC motif. Unrooted *maximum likelihood* phylogeny of ATrx1 homologues defines a monophyletic clade of predicted plastid-targeted proteins with the apicomplexan/chromerid clade branching with sequences from a selected group of heterokont lineages. Outside of this clade are cytosolic proteins that include heterokont paralogues and dinoflagellate proteins. Branch support values for well supported major nodes are bootstraps then Bayesian posterior probabilities. The CXXC motif is highlighted.

To consider the importance of ATrx1 and ATrx2 to plastid function and correlate their presence with complex plastid evolution, we searched for homologues of both sequences in apicomplexans and other eukaryotes with complex plastids. *Tg*ATrx1 homologues were found throughout apicomplexans (coccidia, piroplasms and hemazoans) and were only lacking from *Cryptosporidium* spp., a genus that has lost its plastid. Homologues with greatest similarity to apicomplexan ATrx1s were found in chromerids, dinoflagellates and heterokonts. Molecular phylogenies of ATrx1 sequences resolved the apicomplexans with strong support as a monophyletic group along with chromerids and a subset of heterokont taxa (Fig 2, green shading). This ATrx1 clade excluded dinoflagellate sequences and other heterokonts, including diatoms (Fig 2). Notably, ATrx1 proteins from the clade containing apicomplexans, chromerids and heterokonts all harbor predicted plastid-targeting bi-partite pre-sequences, whereas the outgroup proteins including those of dinoflagellates and more complete representation of heterokonts are all predicted as cytosolic proteins. These data suggest a plastid ATrx1 occurs throughout apicomplexan/chromerid plastid radiation. Further, the phylogeny suggests that a gene duplication within heterokonts allowed for the evolution of the plastid form, and that this was the source of the apicomplexan plastid protein. Plastid ATrx1 has either been lost from dinoflagellates or was never gained, and similarly a cytosolic paralogue in apicomplexans is absent, presumably lost. Curiously, while ATrx1 is common to all apicomplexans with plastids, the protein in piroplasms and hematozoans has lost the otherwise conserved CXXC motif (Fig 2).

ATrx2 is also broadly represented in apicomplexans and other eukaryotes with red-derived plastids, including being encoded in cryptophyte nucleomorphs. Unlike *Tg*ATrx1 homologues, all *Tg*ATrx2 homologues are predicted to occur in plastids. Further, in contrast to ATrx1s, the ATrx2 CXXC motif (CDHC or CEYC) is conserved throughout apicomplexans and chromerids (S1 Fig). The conserved portion of ATrx2 is short and thus ATrx2 phylogenetics were unresolved.

**Fig 3:**
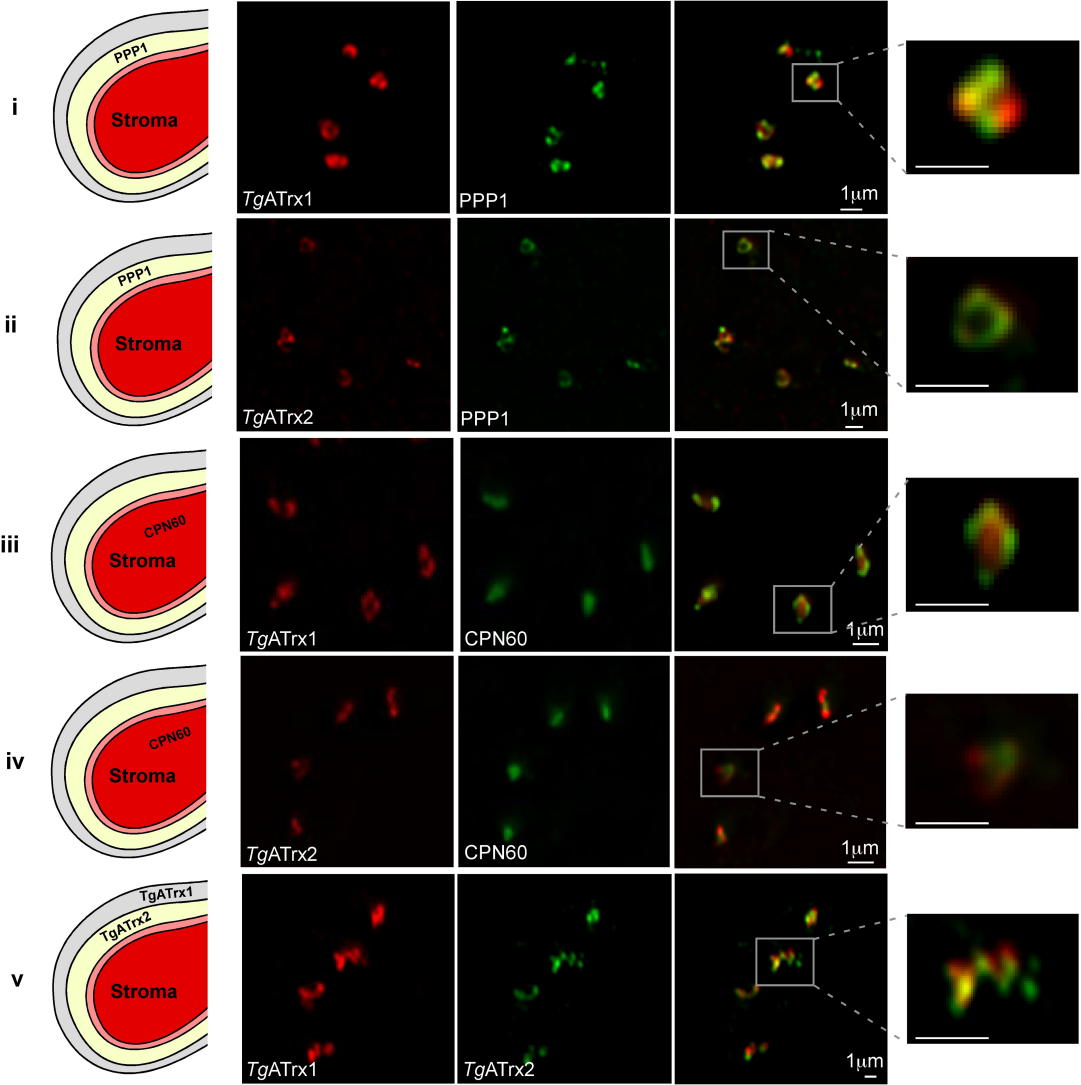
*Tg*ATrx1 and *Tg*ATrx2 show different localization patterns. Fluorescent microscopy of *Tg*ATrx1 (red) co-stained with (i) the PPC marker PPP1, (iii) the luminal marker CPN60 and (v) *Tg*ATrx2 (green); and of *Tg*ATrx2 (red) co-stained with (ii) PPP1 and (iv) CPN60 (green). The images on the right are blow-ups of the regions marked by a white empty square, and their scale bars are 1 μm. The schemes on the left depict the four apicoplast compartments and depict the sub-cellular localization of the markers used for co-staining (PPP1 or CPN60). The illustration in (v) shows the putative sub-compartment localization of *Tg*ATrx1 and *Tg*ATrx2 based on the microscopy and the phylogenetic distribution data, though further work is needed to determine this with certainty. Scale bar, 1 μm.

Our previous studies showed that both *Tg*ATrx1 and *Tg*ATrx2 are residents of the apicoplast periphery. We showed that *Tg*ATrx1 may localize to several peripheral compartments, to vesicles in the cytosol and at low levels to the ER [9], while *Tg*ATrx2 is likely confined to the PPC [10]. To better resolve the location of these two proteins we have employed specific sub-compartmental markers [10] and examined their co-localization using super resolution microscopy. Consistent with our previous observations, signals for both proteins surround the stromal compartment visualized by CPN60 [11]. However, *Tg*ATrx1 and *Tg*ATrx2 only partially co-localize with each other and their staining patterns are different (Fig 3, S2 Fig). *Tg*ATrx2 tightly co-localizes with the PPC marker PPP1, but shows only partial overlap with the outer compartment marker 201270 (the product of *TGME49_201270* previously designated 101270 and 001270 [10]) which lends support to its proposed localization in the PPC (Fig 3, S2 Fig). *Tg*ATrx1 signal shows incomplete overlap with PPP1 but fully overlaps with 201270 and presents additional staining (Fig 3, S2 Fig). The additional signal may correspond to the vesicles previously observed by electron microscopy [9]. Taken together the differences identified in the phylogenetic distribution and localization of the two *Tg*ATrxs suggest distinct functions. The localization further provides additional support to the previous suggestion [10] that *Tg*ATrx2 serves a role that is relevant to the PPC.

### Both *Tg*ATrx1 and *Tg*ATrx2 are essential for growth and their function depends on their CXXC motif

**Fig 4:**
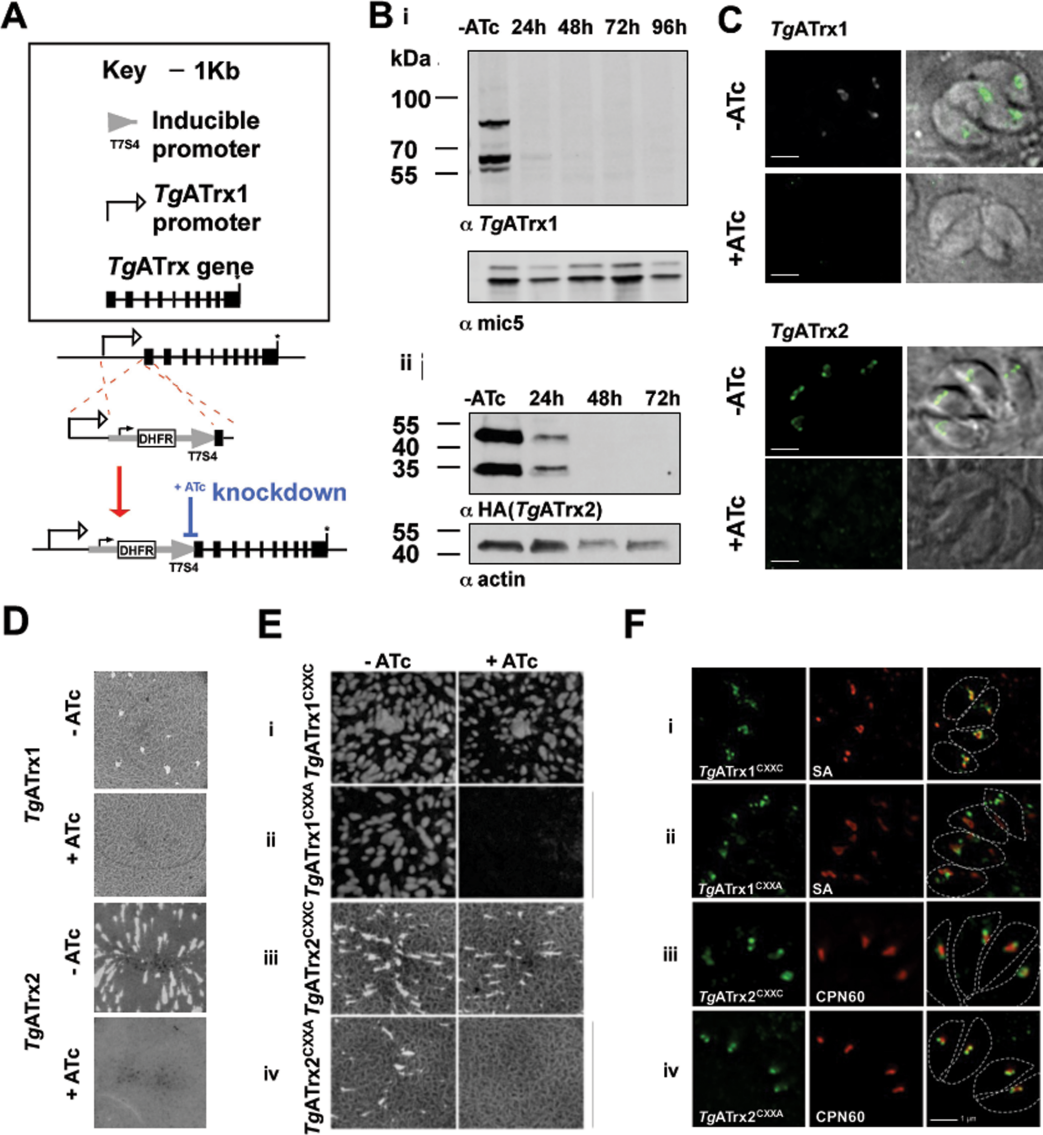
*Tg*ATrx1 and *Tg*ATrx2 are essential and their function requires their CXXC motifs. A. Scheme of the manipulation performed to each of the *Tg*ATrxs loci to replace their promoters with the tetracycline-regulatable promoter. The gene model in the scheme is based on *Tg*ATrx1’s gene model, however the promoter replacement occurred via the same strategy for both *Tg*ATrxs. Black boxes–exons; asterisk–stop codon. B. Western blot analysis of *Tg*ATrx1 expression using anti-*Tg*ATrx1 (i), and endogenously HA-tagged *Tg*ATrx2 using anti-HA (ii), upon ATc treatment. C. Fluorescent microscopy showing *Tg*ATrx1 (anti-*Tg*ATrx1, bottom, green) and *Tg*ATrx2 (anti-HA, top, green) depletion at 48 hours of ATc treatment (+ATc) compared to non-treated control (-ATc). D. Plaque assays performed with TATiΔKu80_PI_ATrx1 (top) and TATiΔKu80_PI_ATrx2-3HA (bottom) with (+) or without (-) ATc. E. Plaque assays performed with TATiΔKu80_PI_ATrx1 constitutively expressing a copy of *Tg*ATrx1^CXXC^ (i) or *Tg*ATrx1^CXXA^ (ii) and with TATiΔKu80_PI_ATrx2-3HA constitutively expressing a copy of *Tg*ATrx2^CXXC^ (iii) or *Tg*ATrx2^CXXA^ (iv). F. Fluorescent microscopy of the localization of *Tg*ATrx1^CXXC^ (i); *Tg*ATrx1^CXXA^ (ii); *Tg*ATrx2^CXXC^ (iii) and *Tg*ATrx2^CXXA^ (iv), all in green, co-stained with Streptavidin (SA) which labels the apicoplast acetyl CoA carboxylase (i, ii) or CPN60 (iii, iv) both in red. White broken line shows parasites’ shapes. Scale bar, 1 μm.

To assess the function of both *Tg*ATrxs in *T*. *gondii* we engineered conditional mutant lines for each of them, in which a tetracycline-regulatable promoter drives the expression of the*Tg*ATrx1 gene or the *Tg*ATrx2 gene as described previously [10] and as illustrated in Fig 4A. For the *Tg*ATrx1 gene this manipulation utilized the TATiΔKu80 line [10] as the parental line. For the *Tg*ATrx2 gene the parental line was a TATiΔKu80 background where the *Tg*ATrx2 coding sequence was endogenously C-terminally tagged with three HA epitope tags as previously described [10]. The resulting lines are named TATiΔKu80_PI_ATrx1 and TATiΔKu80_PI_ATrx2-3HA. Both proteins were downregulated upon addition of anhydrotetracycline (ATc) (Fig 4B and 4C). The previously reported multiple forms of *Tg*ATrx1 [9] were observed by Western blot prior to addition of ATc, and all species fell below the detection levels by 48 hours of ATc treatment (Fig 4Bi). Two forms are detected for *Tg*ATrx2, likely corresponding to the protein before and after cleavage of its targeting presequences, which occurs upon apicoplast import. Both forms fell below the detection level at 48 hours of ATc treatment (Fig 4Bii). To test for growth phenotypes upon *Tg*ATrx1 or *Tg*ATrx2 depletion, plaque assays were performed. In both cases depletion resulted in loss of plaque formation indicating a severe growth defect in the absence of either of these proteins (Fig 4D).

To assess the importance of the putative CXXC active site of each *Tg*ATrx we tested the ability of either wild type *Tg*ATrx, or a mutant form with the second active site cysteine substituted with an alanine (CXXA), to rescue the knockdown phenotypes (see Fig 1B for mechanism). For each regulated line, we constitutively expressed *Tg*ATrx1 or *Tg*ATrx2 minigenes, bearing either the wild type CXXC (*Tg*ATrx1^CXXC^ or *Tg*ATrx2^CXXC^) or a mutated form CXXA (*Tg*ATrx1^CXXA^ or *Tg*ATrx2^CXXA^). For both conditional mutant lines, a constitutively expressed copy with the wild type CXXC rescued the growth phenotype upon ATc treatment, whereas the active site mutant did not (Fig 4E). Immunofluorescence analysis confirmed that the complementing proteins reached the apicoplast (Fig 4F).

ATrx2 sequences have two features that are distinct from classical thioredoxins. First, while thioredoxins are typically small proteins (e.g. human Trx1 and Trx2 are 12 and 18 kDa respectively [12]), ATrx2 orthologues are larger, e.g. *Tg*ATrx2 is ~50kDa with N- and C-terminal extensions to its conserved Trx fold. Second, in classical thioredoxins (e.g. human Trx1 (hTrx1)) the two amino acids of the CXXC motif are typically glycine and proline, and this affects the Trx redox potential [12]. In *Tg*ATrx2 and all its orthologues an acidic and an aromatic residue (CDHC or CEYC) are found (S1 Fig) [10]. To validate that *Tg*ATrx2 can engage in disulphide exchange we tested its activity *in vitro*. Purified recombinant 6xHis-tagged *Tg*ATrx2 (S3 Fig) was incubated with reduced recombinant hTrx1. The resulting samples were alkylated with 4-acetamido-4'-maleimidylstilbene-2,2'-disulfonic acid (AMS), thus adding ~510 Dalton per thiol, resulting in a shift in the migration of proteins with reduced thiols compared to oxidized thiols upon separation by SDS-PAGE. *Tg*ATrx2 showed such a migration shift upon incubation with hTrx1 (S3 Fig), suggesting that the two exchanged disulphides. We further demonstrated that recombinant *Tg*ATrx2 can reduce insulin in an *in vitro* insulin turbidity assay [13] (S3 Fig). These data demonstrate that both *Tg*ATrx1 and *Tg*ATrx2 provide essential functions for *T*. *gondii* growth in culture. The CXXC motifs are essential elements implying that disulphide exchange is key to these functions.

### *Tg*ATrx1 is involved in the control of protein trafficking to the apicoplast

In addition to the typical *Tg*ATrx1 staining at the apicoplast periphery and vesicles around the apicoplast, the mutant TgATrx1^CXXA^ was found in other cellular foci (Fig 4F). This was not the case for *Tg*ATrx2^CXXA^ mutant which maintained the same localization as its wild type parent (Fig 4F). Co-staining with an ER marker imaged by super-resolution microscopy showed that this additional signal is adjacent to and in some cases co-localizing with the ER (S4 Fig). This suggests that the active site mutation enhances ER accumulation beyond the light ER localization observed previously only by electron microscopy [9].

**Fig 5:**
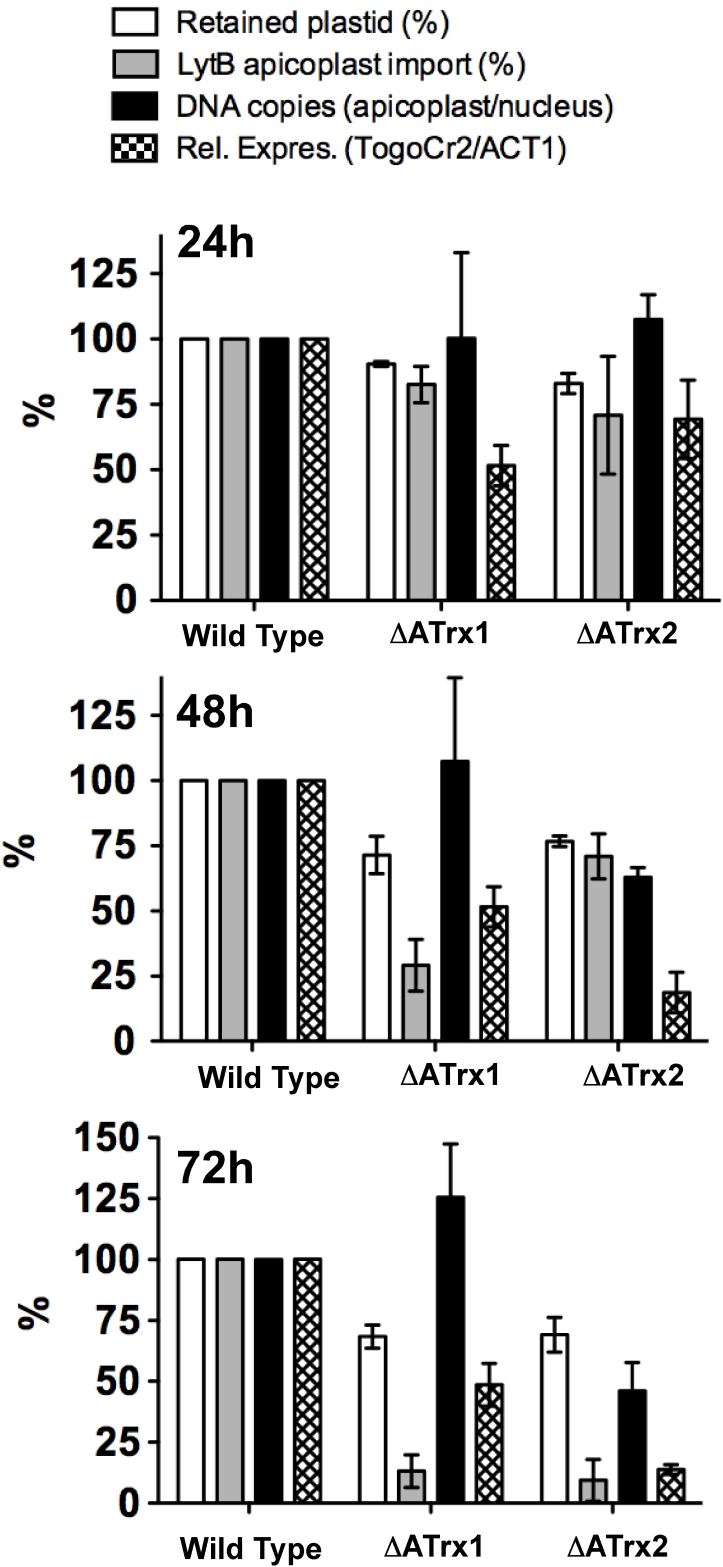
Apicoplast protein import and gene expression are the first biogenesis pathways to reach peak reduction for *TgATrx1* and *TgATrx2* depletion respectively. Summary graphs of apicoplast numbers, LytB protein import, relative genome copy number and mRNA levels measured in both knockdown lines at 24, 48 and 72 hours with ATc. The control (no ATc) data is normalized to 100%. Error-bars are SEM. The corresponding individual graphs are found in S5 Fig.

The altered localisation of *Tg*ATrx1 upon mutation of its CXXC active site raises the possibility that its overall depletion could cause a general protein import defect. Therefore, we investigated the import of known apicoplast stromal (LytB) and peripheral (PPP1) markers under *Tg*ATrx1 depletion using the previously described import assay [10, 14]. The targeting presequences of these proteins are cleaved upon arrival at their destination. When import is compromised, the un-processed precursor accumulates at the expense of the mature form, and this precedes the point of complete organelle loss [10, 14]. Newly synthesized PPP1 showed minor precursor form accumulation at 48 hours of ATc treatment, and by 72 hours no mature protein was detectable (S5 Fig). Likewise, after 72 hours of *Tg*ATrx1 depletion, LytB mature form was undetectable (S5 Fig). We examined apicoplast numbers during *Tg*ATrx1 depletion. Scoring plastids in 100 parasites showed that apicoplast loss was evident at 24 hours and continued gradually (S5 Fig). Note that the complete loss of mature proteins occurs at a time point where 68% of the parasites still have an apicoplast (Fig 5).

In contrast, in the case of *Tg*ATrx2, the mature forms of both LytB and PPP1 were still detected at 72 hours of depletion, despite similar levels of apicoplast loss as seen with *Tg*ATrx1 depletion at this time point (S5 Fig). This suggests that unlike *Tg*ATrx1, when *Tg*ATrx2 is depleted newly synthesized apicoplast proteins can continue to target the organelles that are still intact and reach maturation. These experiments indicated a protein import defect in *Tg*ATrx1-depleted cells that was not seen with depletion of *Tg*ATrx2.

### *Tg*ATrx2 depletion affects apicoplast genome copy number and gene expression

We assessed how other pathways of apicoplast biogenesis and maintenance were affected under *Tg*ATrx2 depletion. We examined apicoplast genome maintenance by measuring the relative copy number of the apicoplast *TogoCr29* gene (which encodes the large subunit rRNA) normalized to the copy number of the nuclear *act1* (*TGGT1_209030*) gene via qPCR [14–16]. At 24 hours of ATc treatment, no significant change was observed of *TogoCr29* compared to the untreated control (S5 Fig). At 48 and 72 hours, *TogoCr29* copies were 60% and 47% of the untreated control. This decrease is more rapid than the observed decrease in apicoplast numbers (Fig 5), suggesting a reduction in *TogoCr29* gene copies in the treated parasites.

Next, we assessed apicoplast transcription. We performed RT-qPCR using total RNA extracted at different time points of ATc treatment. In each reaction, we compared the expression levels of apicoplast-encoded TogoCr29 mRNA to the nucleus-encoded Act1 mRNA. Some decrease in expression (to 89%) was observed at 24 hours, followed by a steep drop to 18% and 13% at 48 and 72 hours (S5 Fig). This reduction largely precedes plastid loss, which was at 69% at 72 hours (Fig 5). These data suggest that apicoplast transcription is decreased upon *Tg*ATrx2 depletion. No tools are currently available to directly measure translation in the apicoplast.

For comparison, we measured apicoplast genome maintenance and gene expression also under *Tg*ATrx1 depletion. At 72 hours of ATc treatment, the apicoplast to nucleus genome copy number ratio was equivalent to that measured in untreated parasites (S5 Fig), indicating no effect on apicoplast genome maintenance. Some decrease in apicoplast gene expression was observed, however, it was significantly milder than that observed following *Tg*ATrx2 depletion (S5 Fig). Fig 5 summarises the measurements of the different pathways at the three time points. The first pathways to reach their lowest values are protein import for *Tg*ATrx1 depletion and gene expression for *Tg*ATrx2 depletion, and this occurs at 48 hours of ATc treatment. At 72 hours, all pathways show defects likely due to secondary effects.

**Fig 6:**
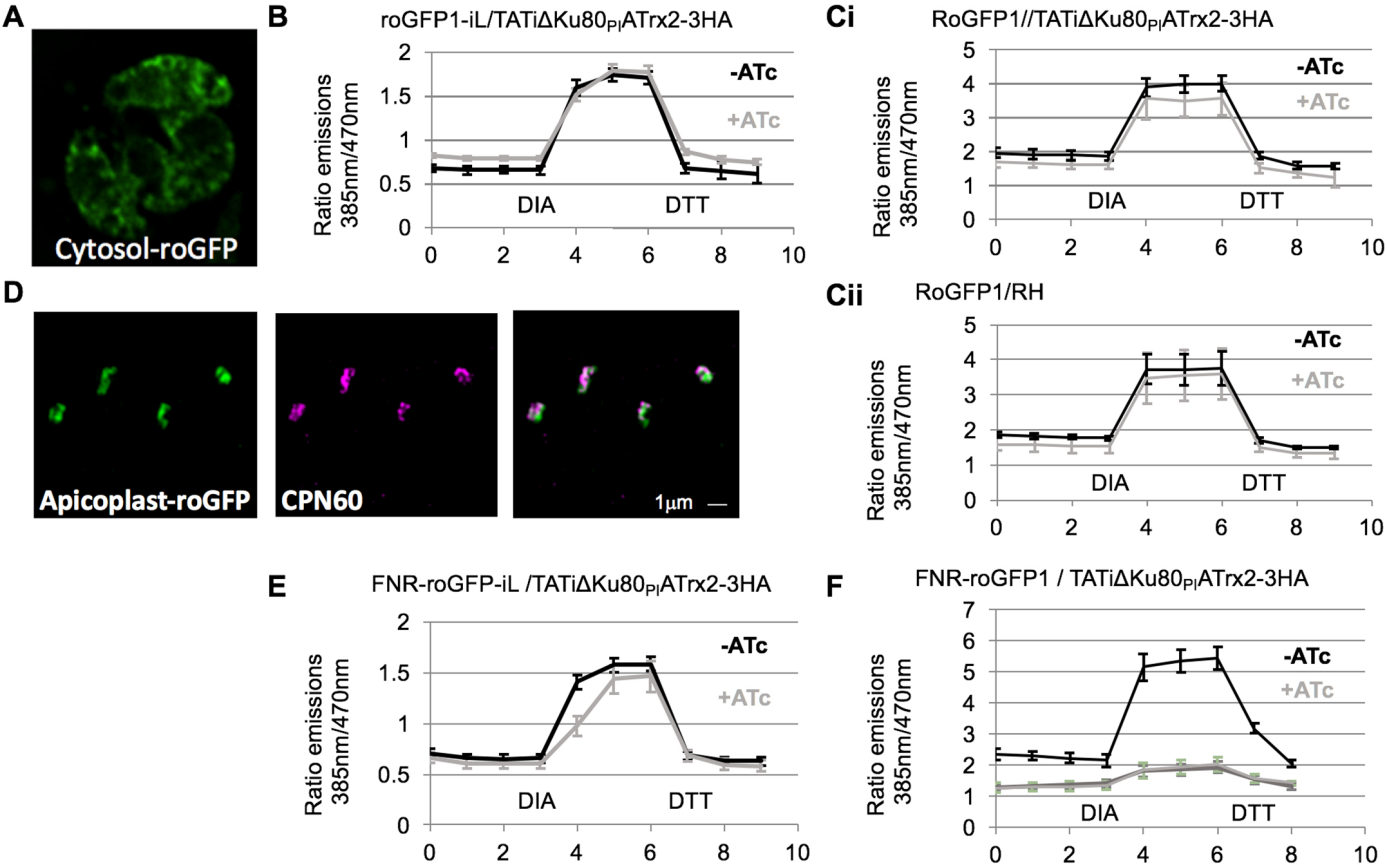
roGFP1 probe suggests apicoplast oxidation upon *TgATrx2* depletion. A. Cytosolic roGFP1 localized in *T*. *gondii*. B/C. Measures of emission ratios (385/470 nm, a higher ratio indicates a more oxidizing environment) of cytosolic roGFPiL (B) and roGFP1 (C) in TATiΔKu80_PI_ATrx2-3HA (B, Ci) or in RH (Cii). D. FNR-roGFP localized to the apicoplast. E/F. Measures of emission ratios of apicoplast roGFPiL (E) and roGFP1 (F) in TATiΔKu80_PI_ATrx2-3HA. X and Y axis show time and ratio of emissions. 10 mM Diamid and 10 mM DTT were added at min 3 and 6. +ATc data is from 72 hours time point except for F, which is 48 hours.

Changes in plastid gene expression can be linked to changes in redox state (reviewed e.g. in [17, 18]). To test the apicoplast stroma redox state under *Tg*ATrx2 depletion we employed the redox sensitive GFP (roGFP) molecules [19, 20] in *Toxoplasma*. These are GFP molecules with two engineered cysteines that can form a disulphide bond, and with two fluorescence excitation peaks at 385 nm and 470 nm. The 385/470 nm ratio increases upon oxidation and decreases upon reduction of roGFP [21, 22]. Since these reporters have not been used in *Toxoplasma* before, we first tested the suitability of two roGFP variants, roGFP1 and roGFP1-iL [21, 22] to report on the parasite’s cytosolic redox state. The two roGFP proteins were transiently expressed and cytosolic localization was confirmed by live fluorescence microscopy (Fig 6A). The change in 385/470 nm ratio was measured in live cells at steady state and during the addition of oxidizing and reducing agents, to assess the dynamic range of each probe. roGFP1-iL is fully reduced in the parasite cytosol at steady state (Fig 6B) and is thus not suitable to assess any potential fluctuation in redox conditions in this compartment. roGFP1 is predominantly, but not fully, reduced (Fig 6Ci), and thus is suitable for measuring redox changes. Neither the parental strain nor the *Tg*ATrx2 conditional knockdown, showed a difference in the cytosolic redox state between ATc treated (72 hours) and non-treated parasites as measured by the roGFP1 assay (Fig 6Ci, ii).

To measure the apicoplast redox state, each roGFP variant was fused to the apicoplast-targeting sequence of ferredoxin NADP reductase (FNR) [23], and apicoplast localization was confirmed by fluorescence microscopy (Fig 6D). FNR-roGFP-iL is fully reduced in the apicoplast stroma and thus not suitable to follow redox fluctuation in this compartment (Fig 6E). In contrast, FNR-roGFP1 showed that the steady state ratios lie within this probe’s dynamic range, making it suitable to measure apicoplast redox changes (Fig 6F). Upon incubation of TATiΔKu80_PI_ATrx2-3HA with ATc, the dynamic range of FNR-roGFP1 changed within 48 hours (Fig 6F). Changes in the dynamic range of a roGFP-based probe due to high compartment oxidation have been observed when another redox probe, Grx1-roGFP2, was used to measure apicoplast redox state in *Plasmodium* parasites in response to drug treatment [24]. We suggest that the change herein is similarly the result of apicoplast oxidation due to *Tg*ATrx2 depletion. Thus, the first identifiable and most prominent phenotype of *Tg*ATrx2 depletion is reduction of apicoplast transcription, followed by a reduction in apicoplast genome maintenance. The onset of transcription defect coincides with a potential redox stress in the apicoplast stroma.

### *Tg*ATrx2 interacts with gene expression and protein translation factors

We reasoned that identification of *Tg*ATrx2 substrates via pull-down might shed further light on its role in controlling apicoplast functions. In some cases, the interaction of Trxs with their substrates can be identified by pull-down of the wild-type (CXXC containing) Trx (e.g. [25]). More transiently associated Trx substrates can be identified using substrate trap mutants. Trap mutants lack the second cysteine of the CXXC motif, which is responsible for resolving the sulfhydryl bonds between Trx and substrate, thus stabilizing the mixed disulphide intermediate between them (illustrated in Fig 1B). This allows pull-down of the covalently coupled substrate along with Trx (e.g. [25, 26]).

**Fig 7:**
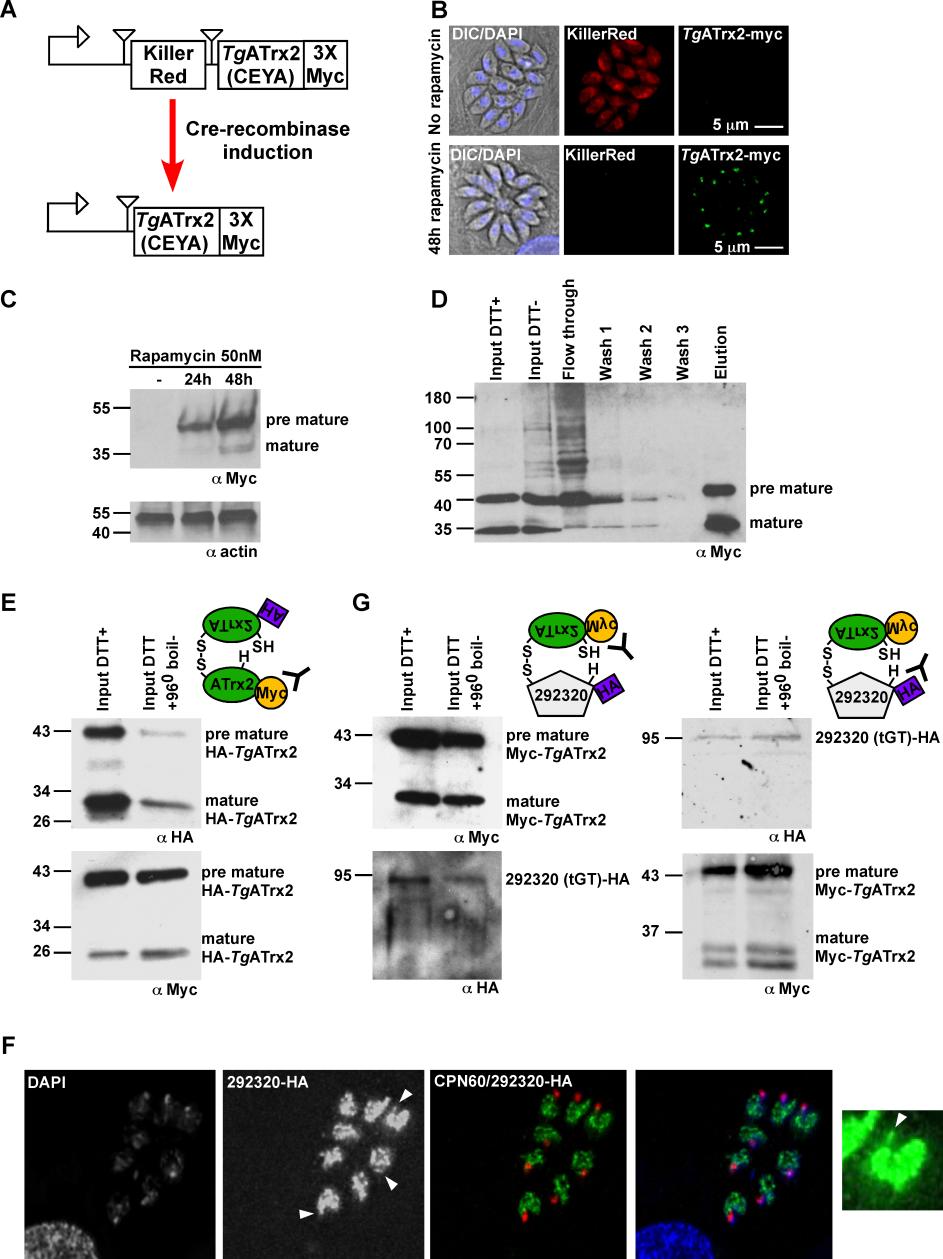
Inducible overexpression of *TgATrx2*^CXXC/A^ and substrate trap identifies gene expression components. A. Scheme of the inducible system. Triangles—LoxP sites. Arrow–promoter. B. Fluorescent microscopy analysis of *TgATrx2*^CXXA^-Myc inducible expression in the presence of 50 nM Rapamycin for 48 hours, showing the switch from killer-red (red) to *TgATrx2*^CXXA^-Myc (green). Scale bar, 5 μm. C/D. Western blot analyses showing the rapamycin induction of *TgATrx2*^CXXA^-Myc (C) and its immunoprecipitation fractions (D) analyzed with anti-Myc antibody. E. Western blot analysis of the co-IP of HA-tagged *TgATrx2* with Myc-tagged *TgATrx2* captured with Myc-Trap beads. F. Fluorescent microscopy of the HA-endogenously tagged TGME49_292320 (green); CPN60 –red; DAPI–blue; Arrowheads highlight apicoplast staining. G. Western blot analysis of the co-IP of HA-tagged TGME49_292320 with Myc-tagged *TgATrx2* captured with Myc-Trap beads (left) and of Myc-tagged *TgATrx2* with HA-tagged TGME49_292320 captured with HA-agarose beads. In D and F, DTT elutes the disulphide bonded partners via reduction and DTT at 96°C treatment elutes all the parasite proteins bound to the beads.

*Tg*ATrx2 is not abundant (http://toxodb.org/toxo/) potentially limiting the identification of its interactors via pull-down at the native levels of expression. To overcome this limitation, we attempted to isolate stable transfectants overexpressing *Tg*ATrx2^CXXA^. This was not successful. Therefore, we engineered an inducible system in which the coding region for *Tg*ATrx2^CXXA^-Myc is separated from a strong promoter by the fluorescent protein KillerRed ORF flanked by LoxP sites (Fig 7A). When integrated into *T*. *gondii* expressing inducible Cre-recombinase [27], rapamycin addition results in KillerRed excision and expression of *Tg*ATrx2-Myc (Fig 7B and 7C). Pull-down experiments were performed in triplicate with parasites expressing inducible *Tg*ATrx2^CXXA^-Myc or *Tg*ATrx2^CXXC^-Myc for 72 hours, and with the parental line expressing no Myc-tagged protein (control for non-specific interaction with the Myc-trap beads).

Fig 7D shows a representative Western blot of one of these pull-down experiments. The whole eluate for each pull-down was analysed by mass spectrometry (S1 Table lists all MS results). Table 1 lists the identified proteins that appeared more than once, with more than two peptides, in the triplicates of *Tg*ATrx2^CXXA^-Myc or *Tg*ATrx2^CXXC^-Myc expressing lines while absent from the triplicates of parental line control; and that were identified with Mascot probability score 20 or above. Ten proteins comply with these criteria: three were pulled down by both forms, one by *Tg*ATrx2^CXXA^-Myc only, and six by *Tg*ATrx2^CXXC^-Myc only. These 10 proteins include a hypothetical protein, a predicted cation efflux transporter and five proteins with predicted functional domains and homology regions shared with factors controlling gene expression and translation (Table 1). Finally, two ribosomal proteins (RPs) were found, RPS25 and RPL4, however, these were previously assigned to cytosolic ribosomes [28] and are likely contaminant in the experiment.

**Table 1:** *Tg*ATrx2 counterpart from pull-down experiments.

Bait	*Tg*ATrx2^CXXC^			*Tg*ATrx2^CXXA^			Information from sequence analysis	Pathway	CXXC	Best BLAST hit from P. falciparum	Information from BLAST, ToxoDB user comments or PlasmoDB literature
Repeat	1	2	3	1	2	3					
**TGME49_310770**	+	+	+				***Tg***ATrx2	This study	Y	PF3D7_0529100	***Tg***ATrx2
**TGME49_249520**	+	+					Hypothetical	N/A	N	N	Homologs only in Toxolasma, Neospora and Hammondia
**TGME49_259250**	+		+				ATP-dependent DNA helicase, RecQ family protein	regulation of gene expression	Y	PF3D7_0918600	PMID: 20016272.
**TGME49_310290**	+		+				regulator of chromosome condensation (RCC1) repeat-containing protein	regulation of gene expression	N	PF3D7_0403100	No functional prediction in ToxoDB, closest homology apicomplexan
**TGME49_210360**	+	+					DEAD (Asp-Glu-Ala-Asp) box polypeptide 41	translation	Y	PF3D7_0527900	Homolog from P. cynomolgi is eIF-4A.
**TGME49_231140**	+	+	+				Ribosomal protein	translation	N	PF3D7_1421200	PMID: 16674839
**TGME49_309120**		+	+				Ribosomal protein	translation	N	PF3D7_0507100	PMID: 24913268
**TGME49_295070**	+	+		+			helicase associated domain (ha2) protein	translation	N	PF3D7_1302700	PMID: 20016272
**TGME49_292320**	+	+	+		+	+	tRNA-guanine transglycosylase	translation	Y	PF3D7_1434100	All apicomplexan homologs have CXXC excluding Cryptosporidium
**TGME49_311720**			+	+	+		BIP	protein folding	N	PF3D7_0917900	PMID 10413671
**TGME49_251630**				+		+	slc30a2	transporter	Y	PF3D7_0715900	

Among the putative interactors of *Tg*ATrx2^CXXC^-Myc was *Tg*ATrx2 itself. We confirmed this interaction by co-immunoprecipitation analysis, using a cell line co-expressing *Tg*ATrx2-HA and *Tg*ATrx2-Myc. Anti-Myc antibody recovered the HA-tagged copy and this interaction was decreased by reduction of the disulphide bonds (Fig 7E). These findings suggest that *Tg*ATrx2 forms oligomers.

To test an additional potential interactor, we selected TGME49_292320, which encodes for a putative tRNA guanine transglycosylase, and which was consistently represented strongly in the pull-down experiments (Table 1), and generated a line where it is endogenously tagged with HA. Immunofluorescence analysis found TGME49_292320 mainly in the parasite nucleus, however with an occasional but reproducible additional apicoplast signal (Fig 7F). Pull-down of *Tg*ATrx2^CXXA^-Myc introduced into this line validated the interaction between the two proteins by Myc-pull-downs recovering HA-tagged TGME49_292320 and *vice versa* (Fig 7G). Taken together *Tg*ATrx2 interactions and the phenotype of its depletion tie its role to the control of the expression of apicoplast genome encoded genes.

## Discussion

Apicomplexan parasites possess an array of redox regulators [29–31], yet the cellular role of many of these regulators has not been studied. Here we address the role of two Trx domain-containing proteins of the apicoplast periphery. Phenotypes resulting from their depletion suggest their involvement in the control of apicoplast protein trafficking and gene expression, revealing two new pathways controlled by redox in these parasites.

### *Tg*ATrx1 controls protein trafficking to the apicoplast

The notion that ATrx1 and ATrx2 have roles in separate pathways is supported by their divergent sequences and phylogenetic histories. The distribution and predicted targeting of ATrx2 orthologues suggest a stable old association with plastids derived from red algae. The universal requirement for transcription regulation control in plastids is consistent with ATrx2’s broad maintenance. Plastid ATrx1, on the other hand, has a more restricted distribution. Dinoflagellates lack the plastid ATrx1, but they also lack one plastid outer compartment as only three membranes surround their plastids. Divergence in plastid protein trafficking pathways between apicomplexans and dinoflagellates is, therefore, consistent with a role of *Tg*ATrx1 in trafficking.

The pattern of inheritance of ATrx1 sequences provided an unexpected perspective on the apicomplexan plastid origin. Plastid gain through endosymbiosis in apicomplexans is argued to have occurred after divergence from ciliates [32], but the source of the plastid remains an open discussion point. Recent plastid phylogenomic analysis suggested that the chromerid/apicomplexan plastid was gained by tertiary endosymbiosis of a non-diatom heterokont [33]. ATrx1 phylogeny indicates that this apicomplexan/chromerid plastid protein was gained from such heterokonts, congruent with this scenario. Lack of plastid ATrx1 paralogues in dinoflagellates could indicate loss of ATrx1 along with loss of one plastid membrane, or that the source of the dinoflagellate plastid is different from that of apicomplexan/chromerids. The loss of the active site CPPC motif in haemosporins and piroplasms is puzzling and might indicate a diverged function of this plastid ATrx1 and participation of alternative Trx proteins in these taxa.

*Tg*ATrx1 depletion results in the reduction of mature apicoplast proteins which starts when *Tg*ATrx1 is fully depleted and reaches below detection levels prior to complete organelle loss (Fig 5, S5 Fig). This response is the same as mutants of different apicoplast import components [10, 11, 14, 34, 35]. We therefore suggest that *Tg*ATrx1 is directly involved in the control of apicoplast protein import. The previous detection of *Tg*ATrx1 within vesicles and in the ER via electron microscopy [9] raises the possibility that it functions in trafficking from the ER to the apicoplast outer membrane. The observed enhanced retention in foci that co-localize with the ER upon active site mutagenesis (S4 Fig) is consistent with this notion. However, regarding the latter observation it cannot be excluded that this retention may be the result of misfolding. It is possible that *Tg*ATrx1 is part of the machinery that separates ER-derived vesicles with apicoplast protein content from vesicles destined elsewhere. Further analysis is required to test this hypothesis.

Two main mechanisms are proposed in the literature whereby disulphide exchange controls protein import: (1) the exchange leads to conformational changes of translocation pore-complex components thus controlling their permeability [36–38]. (2) Disulphide exchange modulates the conformation of the proteins in transit thus affecting their transport competence. For example, in mammalian and yeast cells, members of the protein disulphide isomerase (PDI) family, which contain Trx domains, modulate the folding of secretory proteins in the ER to achieve forms suitable for their onwards transition [7]. It has been suggested that the outermost apicoplast compartment is of ER origin [6]. The second mechanism may apply for *Tg*ATrx1 which might act in an analogous manner to PDIs, engaging with substrates that are flowing from the ER to, and perhaps also through, the outermost apicoplast compartment. *Tg*ATrx1 might assist in controlling their conformational state, thus regulating their onward translocation to the inner compartments. The partial accumulation of *Tg*ATrx1^CXXA^ mutant protein in the ER could be explained by this model whereby trapped *Tg*ATrx1-substrate complexes might be incompetent for forward trafficking from the ER. Put in the context of the above hypothesis of *Tg*ATrx1’s role in vesicle sorting, it is possible that *Tg*ATrx1 escorts proteins destined to the apicoplast within ER-derived vesicles while keeping them in a translocation competent conformational state. A possibility that *Tg*ATrx1 controls import via the first mechanism is also feasible. In that case, loss of translocon permeability upon *Tg*ATrx1^CXXA^ over expression would result in build-up of a protein backlog in the ER.

Redox regulation of protein import via Trx proteins as means to integrate endosymbiotic organelles into the cell metabolism has been discussed for plant chloroplasts and for mitochondria [39]. Our data suggest that redox control is also active in coordinating apicoplast function in the parasite cell.

### *Tg*ATrx2 mediates gene expression via disulphide exchange with translation and transcription components

The first defect detected upon *Tg*ATrx2 depletion is reduction of apicoplast transcription followed by reduction in genome copy number (Fig 5, S5 Fig). Redox regulation of gene expression is well documented. Transcription factors (recently reviewed in [40]), ribosomal proteins, elongation factors and helicases are targets of Trx proteins in different organisms [40–44] including *Plasmodium* [45]. Likewise, ATrx2 pull-downs identified putative substrates with likely roles in gene expression: two candidates have potential roles in transcription (TGME49_259250, 310290) and five in translation (TGME49_210360, 231140, 309120, 295070, 292320) (Table 1).

**Fig 8:**
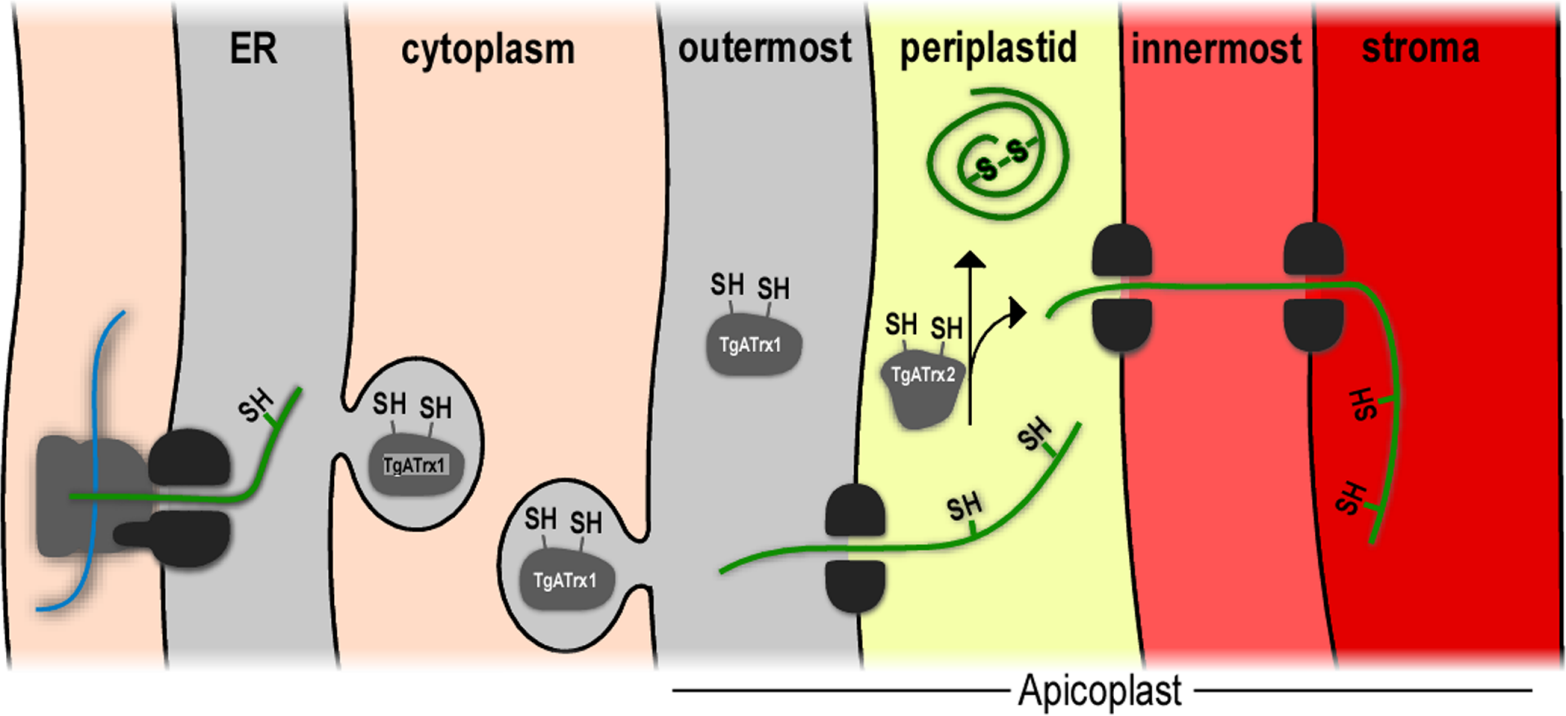
A proposed model of how *TgATrx2* may mediate apicoplast gene expression via disulphide exchange with translation and transcription components as they travel through the PPC. *Tg*ATrx1 and *Tg*ATrx2 are illustrated using dark grey shapes with their thiols shown and are positioned at their putative localization based on previous studies and findings reported herein. The translation and transcription factors whose translocation is proposed to be controlled by *Tg*ATrx2 are represented by green lines. Endomembranes—light grey; Cell cytoplasm–light orange; periplastid compartment–yellow; inner-most compartment–light red; and stromal compartment–red.

A mechanistic model of how the exchange between Trx and gene expression factors controls their function is proposed in some cases. The function of the transcription factors NF-κB is redox modulated in two ways. The DNA binding activity of NF-κB is stimulated by reduction of a disulphide bond via hTrx1 [46]. Additionally, the translocation of NF-κB from the cytosol to the nucleus requires its dissociation from I-κB, which is mediated by hTrx1 [47, 48]. Since we suspect that *Tg*ATrx2 is a PPC resident and transcription occurs in the apicoplast stroma, we hypothesise that it may affect the translocation of its substrates. For example, *Tg*ATrx2 may mediate the folding of its substrates or their interaction with chaperones or escort molecules thus controlling their translocation from the PPC towards the stroma (this hypothesis is illustrated in the model in Fig 8).

One of the *Tg*ATrx2 substrates that we identified (TGME49_292320) is a predicted tRNA guanine transglycosylase (tGT) (Table 1). tGT typically modifies tRNAs with queuine, which affects the efficiency and fidelity of translation [49]. The dual targeting to the nucleus and the apicoplast and reciprocal co-immunoprecipitation (Fig 7) validate the interaction identified by the trap experiments and thus provide support for the role of *Tg*ATrx2 in the control of gene expression via disulphide exchange with translation components.

### Other interactions of *Tg*ATrx2

The pull-down experiments detected inter-molecular disulphide-bonded forms of the wild-type but not substrate-trapping mutant of *Tg*ATrx2 (Table 1), raising the possibility that the oligomerization observed is blocked when *Tg*ATrx2 and its substrate(s) are trapped. This suggests that the interactions with substrates and with other *Tg*ATrx2 molecules occur via the CXXC motif and that they compete. Alternatively, the C-terminal extension may mediate oligomerization in a way that is sensitive to substrate trapping. Dimerization is observed in other Trx proteins. For example, the bacterial PDI DsbC operates as a homodimer and acts both as a chaperone and disulphide bond facilitator. Loss of dimerization reduces both these activities [50].

The pull-down experiments further identified an interaction of *Tg*ATrx2 with the ER chaperone BiP (Table 1). BiP has no cysteines thus the interaction cannot be mediated directly via disulphide bonds. Some PDIs have non-covalent interaction with BiP [25, 51]. It is proposed that PDIs resolve non-native disulphides in substrates that become misfolded and targeted to BiP. For this to take place with *Tg*ATrx2, BiP should target the apicoplast peripheral compartment where *Tg*ATrx2 resides. Like in other organisms, BiP in *Toxoplasma* likely localizes to the ER [52, 53] however its potential presence in compartments of the apicoplast has not been fully assessed.

### Using roGFP to study redox changes in *Toxoplasma*

We have used roGFP molecules for the first time in *Toxoplasma* (Fig 6). roGFP-iL, which has high reduction potential, was fully reduced at steady state in both the cytosol and apicoplast stroma. On the other hand, roGFP1, which has low reduction potential, was partially oxidized in the cytosol and the apicoplast, making it suitable for studies of those compartments. The observation of the apicoplast being reduced corroborates a previous study that used the redox sensitive dimerization of acyl-carrier-protein (ACP) as a redox indicator [54]. A study in *P*. *falciparum*, which used a glutathione-specific sensor, also based on roGFP, similarly revealed a reducing environment in the apicoplast stroma [24]. The change in the dynamic range of FNR-roGFP1 upon *Tg*ATrx2 depletion (Fig 6) was unexpected and may indicate oxidation of the stroma [24]. Since the observed translation defect appeared simultaneously with this change, it is hard to determine the order of events. It is unlikely that *Tg*ATrx2 directly effects the stromal redox state as it does not seem to reside in the stroma (Fig 3iv). The hypothesis that *Tg*ATrx2 depletion affects the transport of translation and transcription components into the stroma, suggests that impaired gene expression directly or indirectly leads to the potential oxidation.

### Summary

We have shown that two apicoplast-specific Trxs are essential for its function and parasite survival. The ATrx2 orthologues have a conserved non-canonical CXXC motif, which is different from hTrx1 and 2 in the identity of the two middle residues. Moreover, the C-terminal extension found in ATrx2 orthologues is not typical for human thioredoxins. These features are conserved in the ATrx2 orthologues from *Plasmodium* spp and other disease-causing apicomplexans, making ATrx2 a particularly attractive drug target candidate. This is supported by findings from the whole genome screen for genes important for growth in blood-stages, that *P*. *berghei* ATrx2 is likely essential [55]. The *in vitro* activity assay established here could be used to develop a platform for inhibitor screening [13]. Furthermore, some anti-malarials currently used in the clinic cause redox stress in the *Plasmodium* apicoplast [24]. Thus, targeting redox-sensitive apicoplast pathways offers exciting prospects for combination therapy using ATrx-targeting drugs.

## Materials and methods

### Cell culture and growth analysis

*T*. *gondii* tachyzoites were grown in human foreskin fibroblasts (HFF, obtained from ATCC, catalogue number #CRC1041). per standard techniques [56]. Where relevant, we added anhydrotetracycline (ATc) to the growth medium at a final concentration of 0.5 μg/mL. For plaque assays, fresh monolayers of HFF were infected with parasites in the presence or absence of 0.5 μg/mL ATc for 7 days. Fixation, staining and visualization were performed as previously described [56].

### Plasmid construction and transfection

Design of **conditional knockdown** vectors: we constructed a vector to guide the insertion of the Tet-inducible promoter [57] between the putative start of ATrx1 and ATrx2 to their putative promoter as described before [10]. Fragments corresponding to the upstream (2877/1047bp) and downstream (1050/582bp) region of the ATrx1/ATrx2 start codons respectively were amplified by PCR using primers 1–8 (S1 Table lists all primers) and inserted into the *NdeI* and the *BglII/AvrII* restriction sites of pDT7S4PPP1.1myc [10] respectively.

Following transfection into TATiΔKu80 or TATiΔKu80_PI_ATrx2-3HA strains, integrants were selected using 1 μM pyrimethamine and confirmed by PCR using primers 9–17 (S1 Table).

**Complementation vectors** were generated by cloning ATrx1 and ATrx2 minigenes into *BglII* and *AvrII* restriction sites within *pUPRT_(TUB)PPP1Ty* [10]. The cysteine to alanine mutation was introduced by site-directed mutagenesis using primers 18–21 (S1 Table). These plasmids were transfected by electroporation into the TATiΔKu80_PI_ATrx1 and TATiΔKu80_PI_ATrx2-3HA lines and stable transgenic lines were selected in 5 μM FUDR.

For **expression of myc-tagged *Tg*ATrx2** in the lines with endogenous HA-tagged *Tg*ATrx2 and TGME49_292320, the *Tg*ATrx2 minigene was cloned between *BglII* and *AvrII* restriction sites within pDT7S4-PPP1-myc [10] using primers 22–23 (S1 Table) creating *pDT7S4-ATrx2-myc*. Stable lines were selected using 1 μM pyrimethamine and confirmed by immunofluorescence with an anti-Myc (Thermo-scientific) antibody.

To create a **switch-on system** the fragment ATrx2-Myc-3’UTR was sub-cloned from the vector *pDT7S4-ATrx2-myc* with primers 24–25 (S1 Table) to the vector *loxP-KillerRed-loxP-YFP* (kindly given by Markus Meissner) [27]. The fragment was inserted between *BglII* and *NotI* restriction sites using primers ATrx2-BglII-loxP-MfeI-F and ATrx2-NotI-R, thereby replacing the original YFP sequence on the vector. Insertion between *BglII* and *NotI* removed the second loxP site on the original vector; therefore a new loxP site was introduced via the forward primer ATrx2-BglII-loxP-MfeI-F. These plasmids were linearized with *ScaI* and transfected by electroporation into the RH DiCre line (kindly given by Markus Meissner) [27]. The stable transgenic lines were selected using mycophenolic acid (25 μg/mL) and xanthine (50 μg/mL) one day after transfection.

To generate **cytosolic roGFP expressing vectors** the roGFP1 and roGFP-iL-KDEL constructs [22] were used as a template with primers 28–29 and 26–27 respectively. For the latter, both the Erp57 signal sequence at the 5' end and the KDEL-coding sequence at the 3' end, used to direct the protein in the ER, were deleted by primer design. The resulting amplicons were each cloned into the *Toxoplasma* TUB8mycGFPMyoATy expression vector between *EcoRI* and *PacI* restriction sites downstream a Myc tag. The new TUB8-myc-roGFP-iL plasmid was transfected by electroporation into the TATiΔKu80_PI_ATrx2-3HA line and the resulting transgenic parasite population was enriched by three rounds of passages in culture plus cell sorting [10] before isolation of stable clones. The new TATiΔKu80_PI_ATrx2-3HA-roGFP1-iL line was confirmed by immunofluorescence performed on isolated clones.

To generate **apicoplast roGFP expressing vectors** the FNR leader sequence was amplified from FNR-RFP [23] template using primers 30–31 and cloned using *EcoRI*/*BstBI* into the *EcoRI* site in each of the two above mentioned pTUB8roGFP vectors.

### Immunoblot analysis

Total parasite lysates (collected at 1500 g, 10 min, RT and lysed in 1x sample buffer), or immunoprecipitation fractions (10^5^ or 10^7^ parasites per lane respectively) were separated by SDS-PAGE and used for immunoblot analyses. After blocking in Odyssey block (LI-COR Biosciences) or in 1X TBS, Tween-0.2%, 5% BSA, blots were probed with: monoclonal mouse anti-ATrx1 11G8 at 1:5000 [9]; mouse anti-HA mAb at 0.1 μg/mL (Covance); rat anti-HA (Sigma-Aldrich, 1:50); Mouse-anti-His (Amersham (GE), 1:1000); rabbit anti-c-Myc (Thermo Scientific, 1:1,000); rabbit anti-Mic5 (gift of Dr. Vern Carruthers, 1:10,000) and anti-actin. This was followed by goat anti-mouse Ig coupled to IRDye 800 (1:10,000, LI-COR) or goat anti-rabbit Ig coupled to IRDye 680 (1:10,000, LI-COR), or goat anti-rabbit or anti-mouse HRP conjugated (Promega, 1:10,000).

### Immunofluorescence assays

Parasites were grown within HFF on coverslips. Immunofluorescence assays were carried out as indicated previously ([14] or [58]). Antibodies and concentrations used were: rat anti-HA (Sigma-Aldrich, 1:50); rabbit anti-c-Myc (Thermo Scientific, 1:1,000); rabbit anti-CSP60 (Reff, 1:1,000); FITC-coupled rat anti-HA (Roche, 3 μg/mL); rabbit anti-IMC1 (gift of Con Beckers, 1:1,000); in Fig 3 the marker for the apicoplast stroma was the naturally biotinylated apicoplast luminal protein acetyl CoA carboxylase revealed by Texas Red coupled-streptavidin (Invitrogen, 1 μg/mL) [59]. Secondary antibodies: Cy2 goat anti-rabbit, Cy3 goat anti-Rabbit, Cy2 goat anti-mouse, Cy3 goat anti-mouse (all Jackson Immuno Research Laboratories, 1:2,000).

### Microscopy

Images were either taken using a Delta Vision microscope, or SuperResolution Structural Illumination Microscopy (SR-SIM). For Deltavision, an RT deconvolution microscope with an Olympus UPlan/Apo 100x 1.35 NA objective was used to view the slides. Images were deconvolved using softWoRx (version 3.5.1) using standard parameters and a conservative ratio algorithm. For SuperResolution stacks of 30–40 images were taken with increments of 0.091 μm in a Zeiss Elyra SuperResolution microscope (Jena, Germany) with a 63x oil immersion objective and an immersion oil with a refractive index of 1.518 (Zeiss, Germany). SuperResolution images were generated using ZEN software (version Zen 2012 SP1, Zeiss, Germany) and processed into their final form using FIJI software [60].

### roGFP assay

TATiΔKu80_PI_ATrx2-3HA-roGFP1-iL or TATiΔKu80_PI_ATrx2-3HA parasites transiently transfected with TUB8rogGFP1 were grown on HFF monolayers on coverslips in the presence or absence of ATc. Cells were rinsed three times with HEPES buffer (20 mM Hepes pH 7.4 containing 130 mM NaCl, 5 mM KCl, 1 mM CaCl_2_, 1 mM MgCl_2_, 10 mM D-glucose), transferred to a microscope chamber and incubated in HEPES buffer at room temperature. A Zeiss Axio Observer A1 inverted microscope equipped with a 40X oil immersionFluor lens was used to image the cells. Fluorescence excitation light was generated by the Colibri illumination system, which alternated the excitation wavelength between 385 and 470 nm. Fluorescence emission at 510 nm was monitored by the computer-controlled AxioVision software. Regions of interest were determined manually including the background. Sequential images were collected every minute and exposure to excitation light was 80–200 ms/image. Cells were oxidized with 1 mM diamid and reduced with 10 mM DTT after 3 and 6 min, respectively. To determine the relative ratio of reduced and oxidized roGFPs, 385/470 nm ratios were determined from the fluorescence intensities of regions of interest after background subtraction.

### Apicoplast protein import assay

Western blot of transiently expressed proteins: TATiΔKu80iATrx1pi or TATiΔKu80_PI_ATrx2-3HA parasites were grown with/out ATc for a given period, then transiently transfected with pBT_LytB or pTUB8-PPP1-HA [14] and let to grow for an additional 24 hours to reach the total desired time of treatment (for example for 72 hours +ATc time point, parasites were grown for 48 hours in ATc, transfected and then grown for an additional 24 hours in ATc). Transfected and treated parasites were collected, total parasite lysate was then separated by SDS-PAGE and blotted using anti-Ty or anti-HA antibodies as described above.

### qRT-PCR

In each experiment, parasites were cultured in triplicates with or without ATc for 24, 48 or 72 hours). Nucleic acids were purified from parasite pellets obtained from fully egressed cultures using the RNeasy (for total RNA) and DNeasy (for gDNA) kits (QIAGEN) and following manufacturer’s instructions. DNA contamination was removed from RNA samples using the Turbo DNA-*free* kit (ThermoFisher Scientific) and samples were reverse transcribed to cDNA using the RETROscript kit (ThermoFisher Scientific). Concentrations of 20 ng of either gDNA or cDNA were then used in each qPCR reaction, which was set up with Power SYBR Green Master Mix (ThermoFisher Scientific) and using 300 nM of each primer. All qPCR reactions were performed using a 7500 Real Time PCR System (Applied Biosystems) using default temperature settings and performing a dissociation step after each run. Relative gene expression was determined using the double Δ Ct method [61]; the same methodology was also used to estimate differences in gene copy numbers as described by Ferreira and colleagues [15].

### Co-immunoprecipitation and mass spectrometry

*Immunopurification*: RH DiCre loxP KillerRed loxP ATrx2 myc parasites were cultured in presence of 50 nM Rapamycin for 72 hours. Parasites were collected, rinsed with PBS supplemented with 20 mM NEM, and lysed for 30 min on ice in lysis buffer (10 mM Tris/HCl pH 8, 150 mM NaCl, 0.5 mM EDTA, 0.5% (v/v) NP-40, 20 mM NEM, 1 mM PMSF) supplemented with protease inhibitor cocktail (Roche). The lysate was span at 20,000 × g for 10 min at 4°C), and the supernatant submitted to denaturation with 0.2% (v/v) SDS for 2 min at 100°C. The sample was pre-cleared with agarose beads (10% slurry) for 30 min at 4°C with rotation. The pre-cleared input was then applied to either Myc-Trap_A agarose beads (Chromotek) or HA epitope Tag Antibody (Pierce) agarose beads overnight at 4°C with rotation. The flow-through was collected and beads were washed three times in dilution buffer (10 mM Tris/HCl pH 8, 150 mM NaCl, 0.5 mM EDTA, 1 mM PMSF) supplemented with protease inhibitor cocktail. Substrates bound to the complex anti-myc-ATrx2-myc were released by incubation with 25 mM DTT for 10 min at RT, and the resulting eluate stored at -20°C. A second elution with 25 mM DTT for 10 min at 95°C allowed detachment of ATrx2-myc from the beads. Input, flow-through, washes and second elution samples were separated by SDS-PAGE and blotted using a rabbit anti-myc antibody (ThermoFisher Scientific, 1:1,000).

*Mass spectrometry*: Proteins were identified using nanoflow HPLC electrospray tandem mass spectrometry (nLC-ESI.MS/MS) at Glasgow Polyomics. Tryptic peptides, generated using the FASP procedure [62] were analyzed as previously described [63]. During result analysis, only peptides with Mascot score of 20 and above (namely the probability that this match might be a random event is 10^−2^ or lower) were included in the analysis.

### Phylogenetics

Alignments were generated using MAFFT [64], manually corrected and ambiguous sites removed. 364 sites were used. Maximum likelihood phylogenies were performed using RAxML v8.1.17 [65] using the best-fit model (LG+G+F) inferred by Prottest 2.4 [66] and 100 resamplings for bootstrap calculations. Bayesian analyses were performed with MrBayes 3.2.6 [67] hosted on the CIPRES Science Gateway webportal (https://www.phylo.org/, last accessed May 24, 2017) [68].

## Supporting information

S1 Table Primers used in this study. The numbers, names and sequences of each primer used in this study are detailed. The purpose column summarizes what was amplified with each pair of primers.
(PDF)

S2 Table Mass spectrometry data from the six ATrx2 substrate trap experiments. Each sheet contains the list of *T*. *gondii* proteins identified in each experiment. For each identified protein the peptides that were found are listed along with their scores.
(XLSX)

S1 Fig Sequence alignment of ATrx2 homologues. Apicomplexans (Api), chromerids (Chr), cryptophytes (Cry), haptophytes, (Hap), heterokonts (Het), and dinoflagellates (Din) including dinoflagellate taxa containing heterokont-derived endosymbionts (Din*). N-terminal protein presequences contain predicted bipartite targeting sequences consistent with plastid location of these proteins. The exceptions are the cryptophyte sequences which lack any N-terminal extension, but are encoded in the nucleomorph of the complex plastid. These proteins are therefore predicted to locate within the plastid periplastidal compartment of the plastid, between membranes two and three of the four. The CXXC motif of ATrx2 (highlighted) is conserved in apicomplexans, chromerids, cryptophytes and some haptophytes although apparently not in the other lineages.
(PDF)

S2 Fig High-resolution microscopy of the co-staining of ATrx1 and ATrx2 with compartmental markers. ATrx1 (top 3 panels, yellow) and of ATrx2 (middle 3 panels, yellow) co-stained with the luminal marker CPN60 (i, iv); with the outer-membrane marker 201270 (ii, v); or with the PPC marker PPP1 (iii, vi) all in magenta. (vii) shows co-staining of ATrx1 (magenta) with ATrx2 (yellow). Each image shows one apicoplast. The graphs show the distribution of both signals over the line depicted in the merge image. For each signal (graph color matches image color) Y-axis shows intensity in pixels, X-axis shows position along the line. Scale 1 μm.
(TIF)

S3 Fig Recombinant expression and isolation of ATrx2 from bacteria. A. His-tagged ATrx2 expression from the pET28a was induced with Isopropyl β-D-1-thiogalactopyranoside (IPTG) for 12 hours at 16°C and analysed by western blot. Wcl–whole cell lysate. Sol–soluble fraction. B. recombinant ATrx2 was isolated from inclusion bodies and refolding, re-solubilised and purified using a Histrap column. Full lysate (load), column flow through, and elution peaks were analysed by western blot. C. 1 μM reduced human thioredoxin (hTrx1) was incubated with different amounts of oxidised recombinant ATrx2 for 15 min at RT. The results were analysed by western blot. * unidentified band (smaller than the expected size of ATrx2); R, fully reduced control; OX, fully oxidised control; The inset isolates only the oxidized and reduced forms to highlight the relevant bands. D. Insulin aggregation/turbidity assay performed by adding 2 μM hTrx1, PDI or ATrx2 to 100 μM human insulin. The graph shows the change in reaction turbidity at 650 nm with time.
(TIF)

S4 Fig Super resolution microscopy of the ER retention of ATrx1_CXXA_. Three sections of the same image of a single parasite transiently expressing Ty1 tagged ATrx1^cxxa^ (magenta) and the ER marker Der1-GFP (yellow). The merge image includes DAPI (blue). The inset and arrow highlight point of co-localization between ATrx1^cxxa^ and Der1-GFP.
(TIF)

S5 Fig Analysis of apicoplast biogenesis pathways upon ATrx1 or ATrx2 depletion. A. Western blot analyses analyzing the maturation of the PPC (PPP1) or luminal (LytB) apicoplast proteins that are transiently expressed for 24 hours at each time point of ATrx1 (i, ii) or ATrx2 (iii, iv) depletion. The bar-graph under each western shows the calculated ratio of intensity between the mature and premature bands at each time point. Each western is a representative example of two repetitions. B. TATiΔKu80_PI_ATrx1 (i) or TATiΔKu80_PI_ATrx2-3HA (ii) parasites were grown in ATc as indicated and plastids were counted based on immunofluorescence signal obtained via staining with anti-CPN60 antibody in 100 parasites for each time point (error bars are calculated from 4 independent experiments). Y-axis shows the percentage of parasites containing a plastid. C. qPCR comparing nuclear encoded gene (*act1*) and apicoplast genome encoded gene (*TogoCr29*) copy numbers at different time points after down regulation of ATrx1 (i) or ATrx2 (ii). D. qRT-PCR analysis performed with total RNA extracts from TATiΔKu80_PI_ATrx1 (i) or TATiΔKu80_PI_ATrx2-3HA (ii) comparing nuclear encoded mRNA (act1) and apicoplast genome encoded mRNA (TogoCr29) expression levels at different time points after down regulation of ATrx proteins. All the panels show data from parasites grown in the absence of ATc (-ATc) or presence of ATc for 24 (+24h), 48 (+48h) and 72 (+72h) hours. In panel C and D the data was normalized such that copy numbers from each genome from the no ATc treatment sample is 1. All error-bars are SEM.
(TIF)

S1 Materials and Methods Includes detailed description of the in vitro insulin turbidity assay and the Recombinant protein expression and isolation. (PDF)
