# Supplementary material for: Two essential Thioredoxins mediate apicoplast biogenesis, protein import, and gene expression in Toxoplasma gondii
Source: PLoS Pathog. 2018 Feb 22;14(2):e1006836. doi: 10.1371/journal.ppat.1006836 (PMC5823475; doi:10.1371/journal.ppat.1006836)
Supplement: S1 Table — The numbers, names and sequences of each primer used in this study are detailed. The purpose column summarizes what was amplified with each pair of primers. (PDF) [file ppat.1006836.s001.pdf]

Table S1

Primer number	Primer name	Primer sequence	Purpose
1	Atrx_F_Bcl	GGtgatcaATGGCGCCTGACAGTTGCAGACCGG	5' flank for promoter insertion, ATrx1
2	Atrx_R_Avr	GGcctaggTTCGGGGACCGCCAAAGAATTTCTTTCC	
3	Atrx_3'R_Avr	GGGcctaggTCCTCCTCTTCGTGAGGCTTCTCGGC	3' flank for promoter insertion, ATrx1
4	Atrx_3'F_Bcl	GGGtgatcaATGGCGCCTGACAGTTGCAGACC	
5	ATrx2PI_Ase_F	gggATTAATGGCTAGGAAGGGTATCACAACGG	5' flank for promoter insertion, ATrx2
6	ATrx2PI_Ase_R	cccATTAATGGAAGTCGTTCTGGGAATACGC	
7	Atrx2_3'R_Avr	GGcctaggGTCCTTTCGTTTCGAGCGATCTGC	3' flank for promoter insertion, ATrx2 (the whole minigene)
8	Atrx2_3'F_Bgl	GGGagatctATGATGAAGCAATCCTTTTCCCGG	
9	AtrxTestR	CCGAGAGTCTCCTGCGTCCACTCCG	Test primer to confirm ATrx1 locus change
10	AtrxTest2F	GCCCCTGGCGAGCGCCGTGGATGG	
11	ATrx2n KOtest1	GCGCCTTCCTTCCCTGTCCCG	Test primer to confirm ATrx2 locus change
12	ATrx2PItestF	CCAGGGACAGAGCGTCGTATGC	
13	ATrx2Ftest	GCAAACGCACAGACAGGCGCAC	
14	ATrx2Rtest2	GTGCGCCTGTCTGTGCGTTTGC	
15	ATrx2Ftest3	GCGACGATAGCTCGATTTTACCGTCCGG	
16	T7S4_F	CGCCTTGGCGAATGTTCATGAC	Test primer on the modification cassette
17	DHFR_cassette_R	CGCACGGCAGTCAGATAACAGGTGTA	
18	ATrx1WCPPAs	CTCTTGGTGTCCGCCTGCTCAGGCGTTTAC	cysteine to Alanine mutagenesis, ATrx1
19	ATrx1WCPPAas	GTAAACGCCTGAGCAGGCGGACACCAAGAG	
20	ATrx2_CtoA_F	GTTGGGGAGGAATGCGAATACgct AGAAGAATGGAGCCTGTCGAG	cysteine to Alanine mutagenesis, ATrx2
21	ATrx2_CtoA_R	CTCGACAGGCTCCATTCTTCTagc GTATTCGCATTCCTCCCCAAC	
22	ATrx2atgBglIIF	GGGagatctATGATGAAGCAATCCTTTTCCCGG	Clone ATrx2 minigene into FUDR selectable complementation vector and into pDT7S4 to express Myc tag for reproduced co-IP
23	ATrx2AvrIIR	GGcctaggGTCCTTTCGTTTCGAGCGATCTGC	
24	ATrx2-BglII-loxP-MfeI-F	AGATCTATAACTTCGTATAGCATACATTATACGAAG TTATCAATTGATGATGAAGCAATCCTTTTCCCGGCTGGC	Rapamycin turn-on system
25	ATrx2-NotI-R	GCGGCCGCTCTAGAACTAGCTAGTGGATCCCCCTCC	
26	roGFPiL-EcoRI-F	gggccGAATTCATGGTGTCCAAGGGCGAGG	pTUB8 roGFP 1iES
27	roGFPiL-PacI-R	gggccTTAATTAATCACTTGTACAGCTCGTCCATGC	
28	roGFP1-EcoRI-F	gggccGAATTCATGGTGAGCAAGGGCGAGGAGC	pTUB8 roGFP 1
29	roGFP1-PacI-R	gggccTTAATTAATTACTTGTACAGCTCGTCCATGCCG	
30	FNR-EcoRI-F	ggggGAATTCATGGTTCGGGGCATCCGTCC	FNR-roGFP
31	FNR-BStBI-R	ggggTTCGAAGGATGTTTGGTCGGTCGGGG	FNR-roGFP
32	ATrx2 Fwd EcoR1	CGCGAATTCCTCACAAGCCCGTCTCCCGGTCCG	ATrx2 into pET28a for bacterial expression
33	ATrx2 Rev XhoI	CGCCTCGAGCTAGTCCTTTCGTTTCGAGCGATC	
34	52_292320_LIC_F	TACTTCCAATCCAATTTAATGCGGACCTCTGGAGCTCC	TGME49_292320 endogenous tagging
35	53_292320_LIC_R	TCCTCCACTTCCAATTTTAGCGGCCGTGATCGCTCTGCA	
36	TogoCr29qPCR-F	GCCCGTACTAAAACTGACACAAG	Amplification of TogoCr29 by qPCR
37	TogoCr29qPCR-R	AGGCATCCTTTATCCCGAAG	
38	ACT1qPCR-F	GGGACGACATGGAGAAAATC	Amplification of ACT1 by qPCR
39	ACT1qPCR-R	AGAAAGAACGGCCTGGATAG	
