# Supplementary material for: Two essential Thioredoxins mediate apicoplast biogenesis, protein import, and gene expression in Toxoplasma gondii
Source: PLoS Pathog. 2018 Feb 22;14(2):e1006836. doi: 10.1371/journal.ppat.1006836 (PMC5823475; doi:10.1371/journal.ppat.1006836)
Supplement: S1 Fig — Apicomplexans (Api), chromerids (Chr), cryptophytes (Cry), haptophytes, (Hap), heterokonts (Het), and dinoflagellates (Din) including dinoflagellate taxa containing heterokont-derived endosymbionts (Din*). N-terminal protein presequences contain predicted bipartite targeting sequences consistent with plastid location of these proteins. The exceptions are the cryptophyte sequences which lack any N-terminal extension, but are encoded in the nucleomorph of the complex plastid. These proteins are therefore predicted to locate within the plastid periplastidal compartment of the plastid, between membranes two and three of the four. The CXXC motif of ATrx2 (highlighted) is conserved in apicomplexans, chromerids, cryptophytes and some haptophytes although apparently not in the other lineages. (PDF) [file ppat.1006836.s003.pdf]

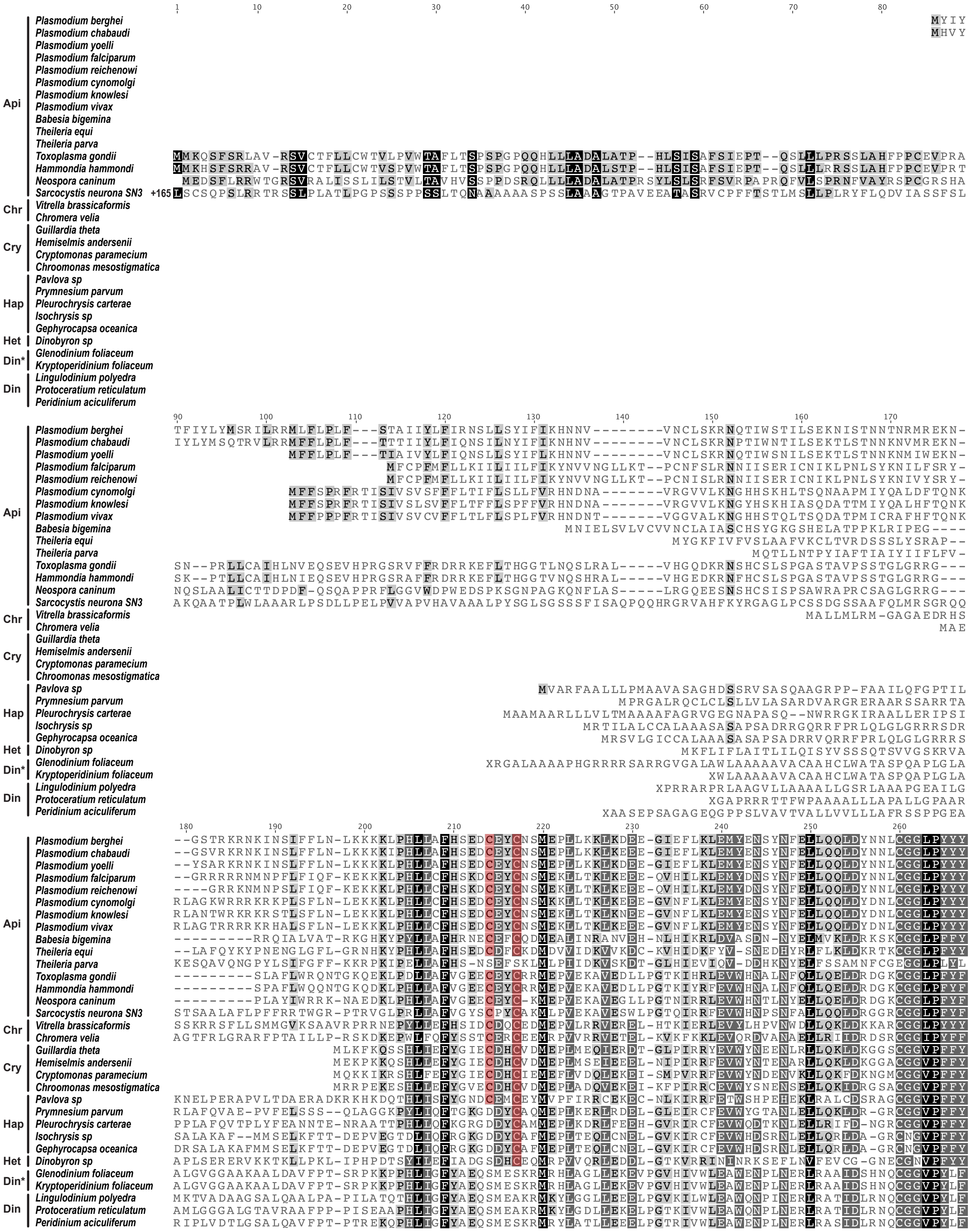

Api
Plasmodium berghei
Plasmodium chabaudi
Plasmodium yoelli
Plasmodium falciparum
Plasmodium reichenowi
Plasmodium cynomolgi
Plasmodium knowlesi
Plasmodium vivax
Babesia bigemina
Theileria equi
Theileria parva
Toxoplasma gondii
Hammondia hammondi
Neospora caninum
Sarcocystis neurona SN3
+165
Chr
Vitrella brassicaformis
Chromera velia
Cry
Guillardia theta
Hemiselmis andersenii
Cryptomonas paramecium
Chroomonas mesostigmatica
Hap
Pavlova sp
Prymnesium parvum
Pleurochrysis carterae
Isochrysis sp
Gephyrocapsa oceanica
Het
Dinobyron sp
Din*
Glenodinium foliaceum
Kryptoperidinium foliaceum
Din
Lingulodinium polyedra
Protoceratium reticulatum
Peridinium aciculiferum

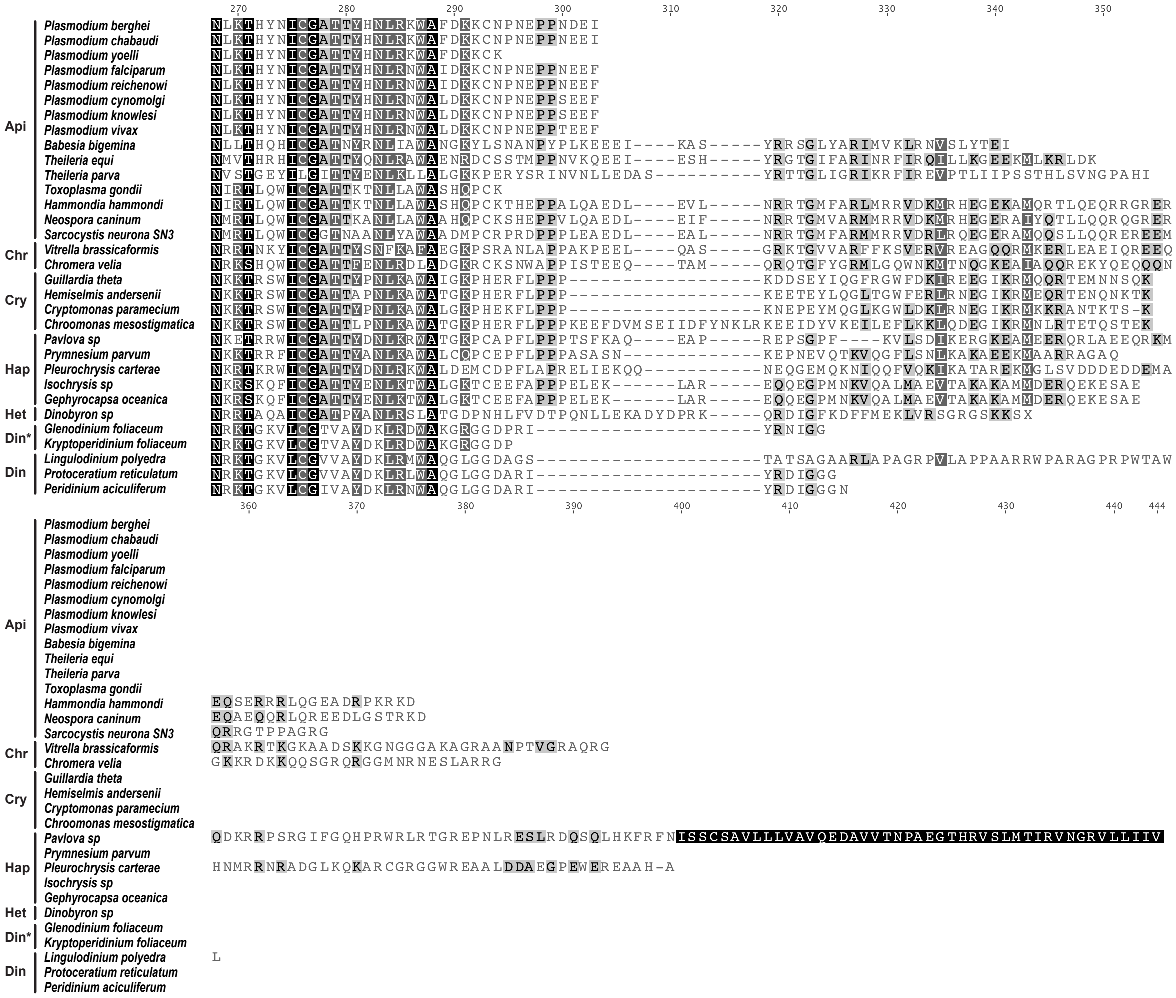

270 280 290 300 310 320 330 340 350
Api
Plasmodium berghei NLKTHYNICGATTYHNLRKWAFDKKCNPNEPPNDEI
Plasmodium chabaudi NLKTHYNICGATTYHNLRKWAFDKKCNPNEPPNEEI
Plasmodium yoelli NLKTHYNICGATTYHNLRKWAFDKKCK
Plasmodium falciparum NLKTHYNICGATTYHNLRNWAIDKKCNPNEPPNEEF
Plasmodium reichenowi NLKTHYNICGATTYHNLRNWAIDKKCNPNEPPNEEF
Plasmodium cynomolgi NLKTHYNICGATTYHNLRNWALDKKCNPNEPPSEEF
Plasmodium knowlesi NLKTHYNICGATTYHNLRNWALDKKCNPNEPPSEEF
Plasmodium vivax NLKTHYNICGATTYHNLRNWALDKKCNPNEPPTEEF
Babesia bigemina NLLTHQHICGATNYRNLIAWANGKYLSNANPYPLKEEEI----KAS-----YRRSGLYARIMVKLRNVSLYTEI
Theileria equi NMVTHRHICGATTYQNLRAWAENRDCSSTMPPNVKQEEI----ESH-----YRGTGIFARINRFIRQILLKGEEKMLKRLDK
Theileria parva NVSTGEYILGITTYENLKLLALGKKPERYSRINVNLLEDAS---------YRTTGLIGRIKRFIREVPTLIIPSSTHLSVNGPAHI
Toxoplasma gondii NIRTLQWICGATTKTNLLAWASHQPCK
Hammondia hammondi NIRTLQWICGATTKTNLLAWASHQPCKTHEPPALQAEDL----EVL-----NRRTGMFARLMRRVDKMRHEGEKAMQRTLQEQRRGRER
Neospora caninum NMRTLQWICGATTKANLLAWAAHQPCKSHEPPVLQAEDL----EIF-----NRRTGMVARMMRRVDKMRHEGERAIYQTLLQQRQGRER
Sarcocystis neurona SN3 NMRTLQWICGGTNAANLYAWAADMPCRPRDPPLEAEDL----EAL-----NRRTGMFARMMRRVDRLRQEGERAMQQSLLQQREREEM
Chr
Vitrella brassicaformis NRRTNKYICGATTYSNFKAFAEGKPSRANLAPPAKPEEL----QAS-----GRKTGVVARFFKSVERVREAGQQRMKERLEAEIQREEQ
Chromera velia NRKSHQWICGATTFENLRDLADGKRCKSNWAPPISTEEQ----TAM-----QRQTGFYGRMLGQWNKMTNQGKEAIAQQREKYQEQQQN
Cry
Guillardia theta NKKTRSWICGATTYPNLKAWAIGKPHERFLPPP-----------------KDDSEYIQGFRGWFDKIREEGIKRMQQRTEMNNSQK
Hemiselmis andersenii NKKTRSWICGATTAPNLKAWATGKPHERFLPPP-----------------KEETEYLQGLTGWFERLRNEGIKRMEQRTENQNKTK
Cryptomonas paramecium NKKTRSWICGATTYPNLKAWALGKPHEKFLPPP-----------------KNEPEYMQGLKGWLDKLRNEGIKRMKKRANTKTS-K
Chroomonas mesostigmatica NKKTRSWICGATTLPNLKAWATGKPHERFLPPPKEEFDVMSEIIDFYNKLRKEEIDYVKEILEFLKKLQDEGIKRMNLRTETQSTEK
Hap
Pavlova sp NKETRRWICGATTYDNLKRWATGKPCAPFLPPPTSFKAQ----EAP-----REPSGPF----KVLSDIKERGKEAMEERQRLAEEQRKM
Prymnesium parvum NKKTRRFICGATTYANLKAWALCQPCEPFLPPPASASN-------------KEPNEVQTKVQGFLSNLKAKAEEKMAARRAGAQ
Pleurochrysis carterae NKRTKRWICGATTYDNLRKWALDEMCDPFLAPRELIEKQQ-----------NEQGEMQKNIQQFVQKIKATAREKMGLSVDDDEDDEMA
Isochrysis sp NKRSKQFICGATTYENLKTWALGKTCEEFAPPPELEK------LAR-----EQQEGPMNKVQALMAEVTAKAKAMMDERQEKESAE
Gephyrocapsa oceanica NKRSKQFICGATTYENLKTWALGKTCEEFAPPPELEK------LAR-----EQQEGPMNKVQALMAEVTAKAKAMMDERQEKESAE
Het
Dinobyron sp NRRTAQAICGATPYANLRSLATGDPNHLFVDTPQNLLEKADYDPRK-----QRDIGFKDFFMEKLVRSGRGSKKSX
Din*
Glenodinium foliaceum NRKTGKVLCGTVAYDKLRDWAKGKRGGDPRI---------------------YRNIGG
Kryptoperidinium foliaceum NRKTGKVLCGTVAYDKLRDWAKGKRGGDP
Din
Lingulodinium polyedra NRKTGKVLCGVVAYDKLRMWAQGLGGDAGS---------------------TATSAGAARLAPAGRPVLAPPAARRWPARAGPRPWTAW
Protoceratium reticulatum NRKTGKVLCGVVAYDKLRLWAQGLGGDARI---------------------YRDIGG
Peridinium aciculiferum NRKTGKVLCGIVAYDKLRNWAQGLGGDARI---------------------YRDIGGGN
360 370 380 390 400 410 420 430 440 444
Api
Plasmodium berghei
Plasmodium chabaudi
Plasmodium yoelli
Plasmodium falciparum
Plasmodium reichenowi
Plasmodium cynomolgi
Plasmodium knowlesi
Plasmodium vivax
Babesia bigemina
Theileria equi
Theileria parva
Toxoplasma gondii
Hammondia hammondi EQSERRRLQGEADRPKRKD
Neospora caninum EQAEQQRLQREEDLGSTRKD
Sarcocystis neurona SN3 QRRGTPPAGRG
Chr
Vitrella brassicaformis QRAKRTKGKAADSKKGNGGGAKAGRAANPTVGRAQRG
Chromera velia GKKRDKKQQSGRQRGGMNRNESLARRG
Cry
Guillardia theta
Hemiselmis andersenii
Cryptomonas paramecium
Chroomonas mesostigmatica
Hap
Pavlova sp QDKRRPSRGIFGQHPRWRLRTGREPNLRESLRDQSQLHKFRFNISSCSAVLLLVAVQEDAVVTNPAEGTHRVSLMTIRVNGRVLLIIV
Prymnesium parvum
Pleurochrysis carterae HNMRRNRADGLKQKARCGRGGWREAALDDAEGPEWEREAAH-A
Isochrysis sp
Gephyrocapsa oceanica
Het
Dinobyron sp
Din*
Glenodinium foliaceum
Kryptoperidinium foliaceum
Din
Lingulodinium polyedra L
Protoceratium reticulatum
Peridinium aciculiferum
