# Supplementary figures and images for: Two essential Thioredoxins mediate apicoplast biogenesis, protein import, and gene expression in Toxoplasma gondii
Source: PLoS Pathog. 2018 Feb 22;14(2):e1006836. doi: 10.1371/journal.ppat.1006836 (PMC5823475; doi:10.1371/journal.ppat.1006836)

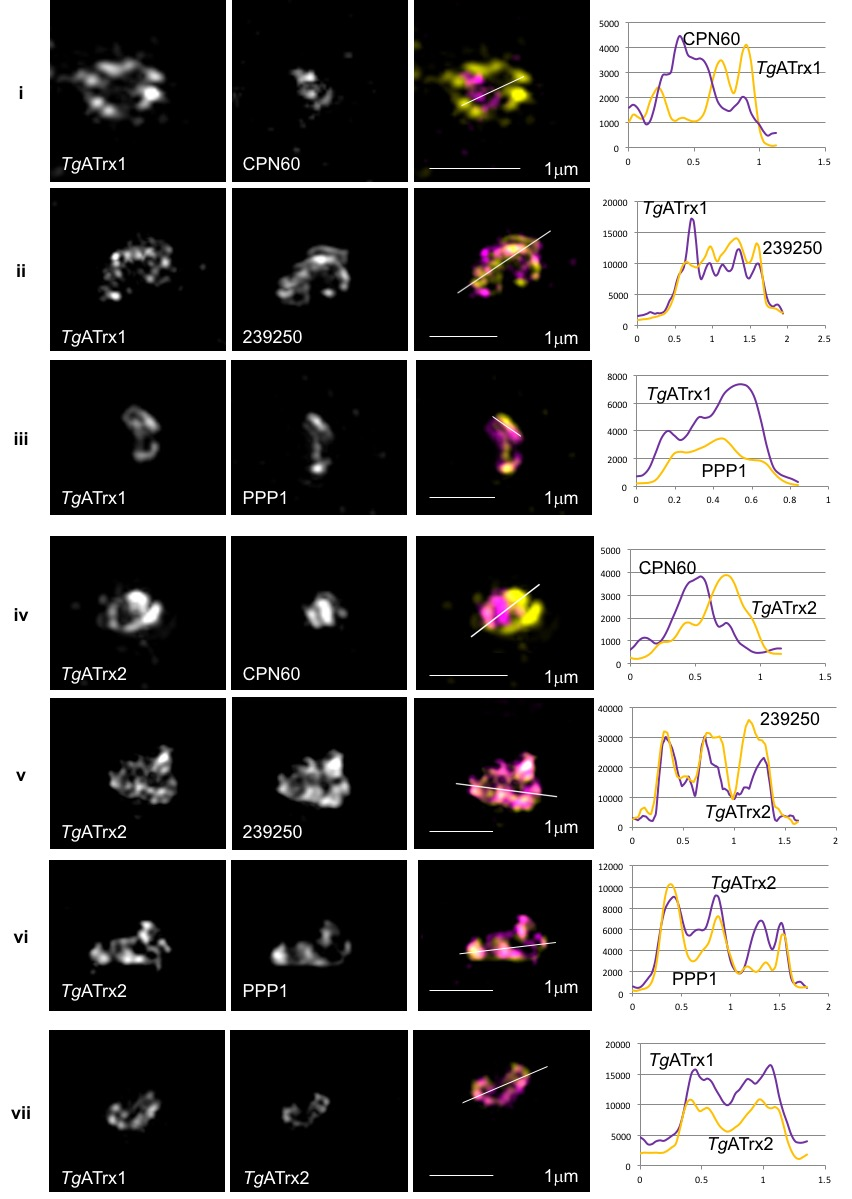

Supplement: S2 Fig — ATrx1 (top 3 panels, yellow) and of ATrx2 (middle 3 panels, yellow) co-stained with the luminal marker CPN60 (i, iv); with the outer-membrane marker 201270 (ii, v); or with the PPC marker PPP1 (iii, vi) all in magenta. (vii) shows co-staining of ATrx1 (magenta) with ATrx2 (yellow). Each image shows one apicoplast. The graphs show the distribution of both signals over the line depicted in the merge image. For each signal (graph color matches image color) Y-axis shows intensity in pixels, X-axis shows position along the line. Scale 1 μm. (TIF) [file ppat.1006836.s004.tif]

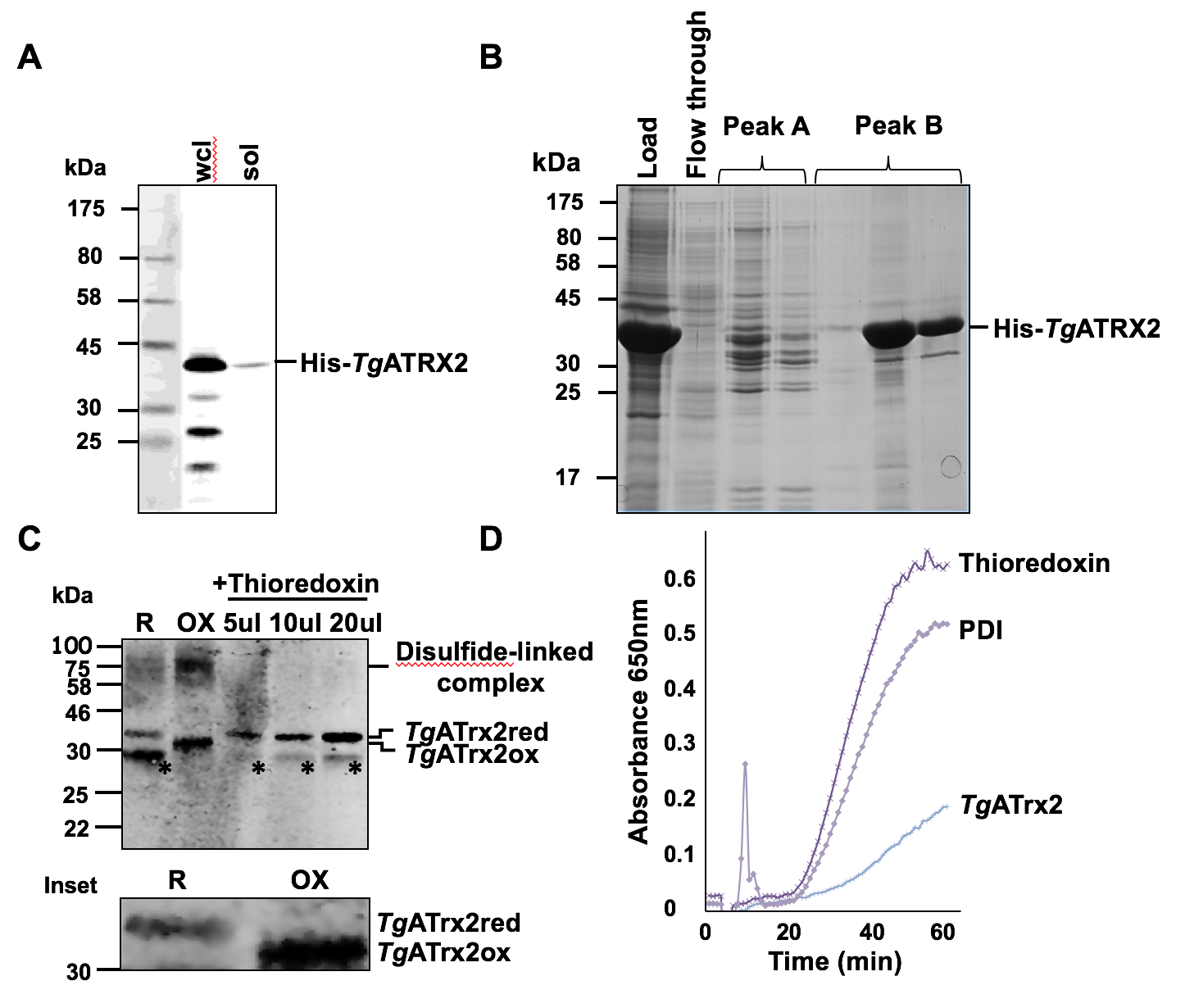

Supplement: S3 Fig — A. His-tagged ATrx2 expression from the pET28a was induced with Isopropyl β-D-1-thiogalactopyranoside (IPTG) for 12 hours at 16°C and analysed by western blot. Wcl–whole cell lysate. Sol–soluble fraction. B. recombinant ATrx2 was isolated from inclusion bodies and refolding, re-solubilised and purified using a Histrap column. Full lysate (load), column flow through, and elution peaks were analysed by western blot. C. 1 μM reduced human thioredoxin (hTrx1) was incubated with different amounts of oxidised recombinant ATrx2 for 15 min at RT. The results were analysed by western blot. * unidentified band (smaller than the expected size of ATrx2); R, fully reduced control; OX, fully oxidised control; The inset isolates only the oxidized and reduced forms to highlight the relevant bands. D. Insulin aggregation/turbidity assay performed by adding 2 μM hTrx1, PDI or ATrx2 to 100 μM human insulin. The graph shows the change in reaction turbidity at 650 nm with time. (TIF) [file ppat.1006836.s005.tif]

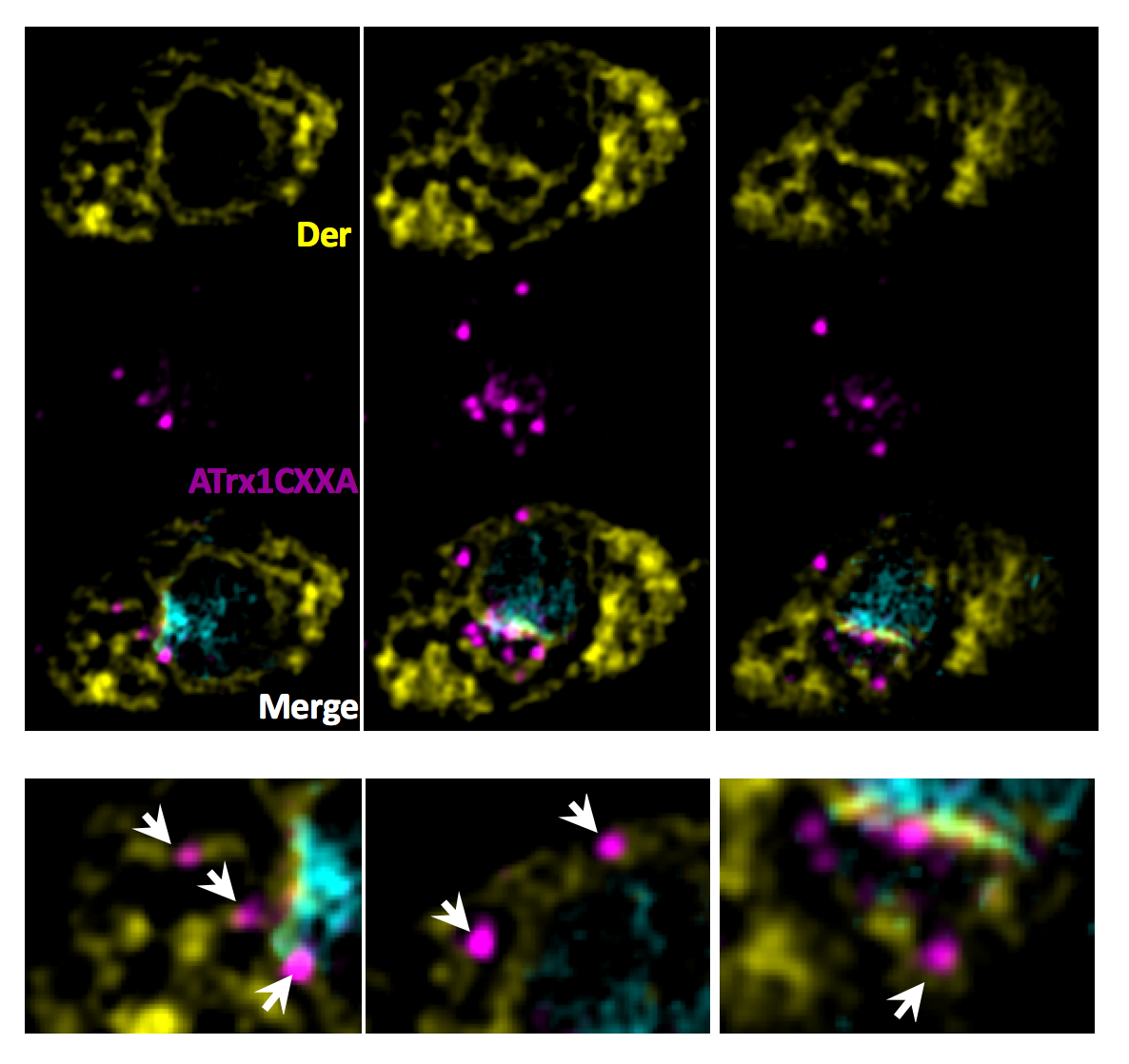

Supplement: S4 Fig — Three sections of the same image of a single parasite transiently expressing Ty1 tagged ATrx1cxxa (magenta) and the ER marker Der1-GFP (yellow). The merge image includes DAPI (blue). The inset and arrow highlight point of co-localization between ATrx1cxxa and Der1-GFP. (TIF) [file ppat.1006836.s006.tif]

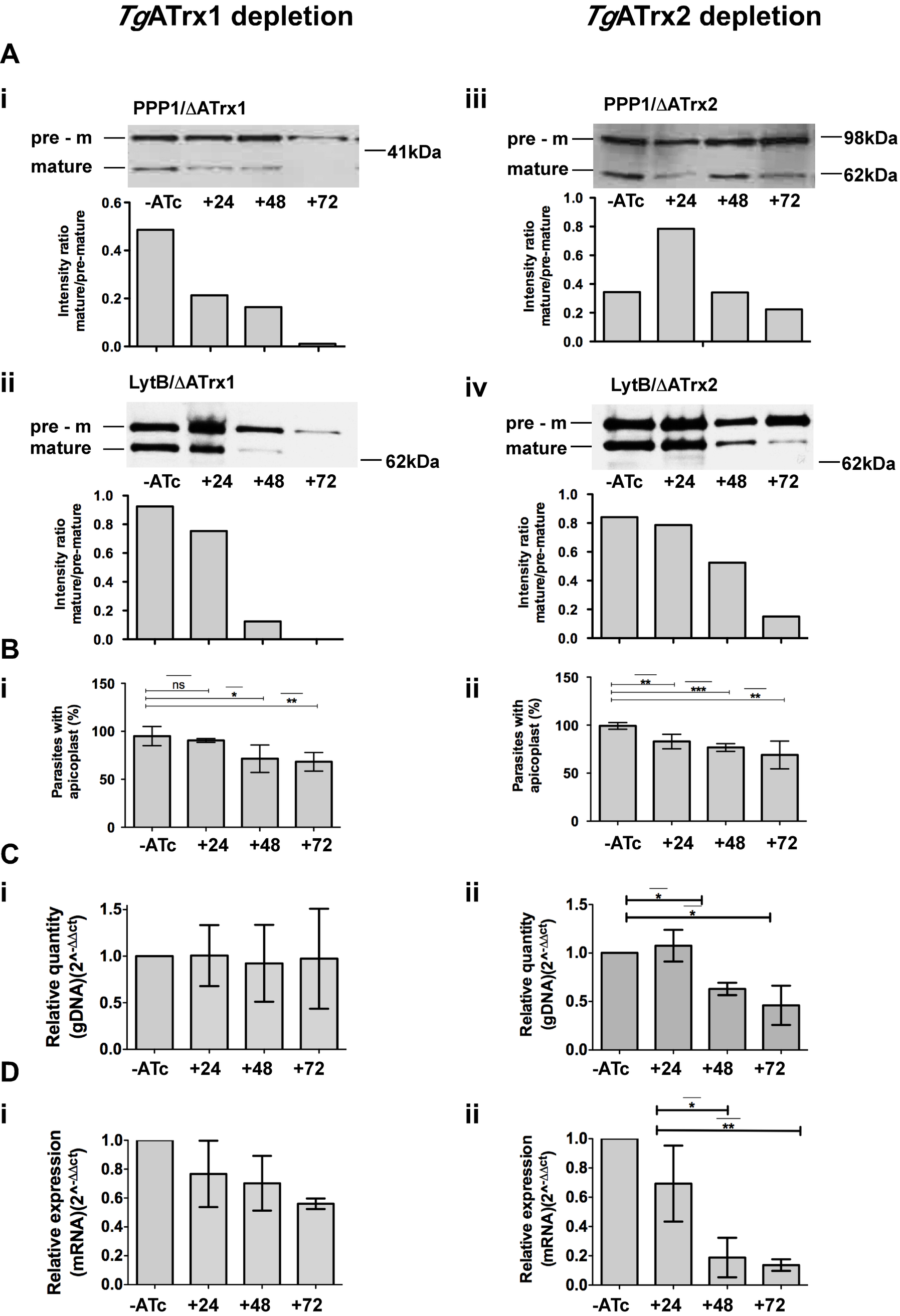

Supplement: S5 Fig — A. Western blot analyses analyzing the maturation of the PPC (PPP1) or luminal (LytB) apicoplast proteins that are transiently expressed for 24 hours at each time point of ATrx1 (i, ii) or ATrx2 (iii, iv) depletion. The bar-graph under each western shows the calculated ratio of intensity between the mature and premature bands at each time point. Each western is a representative example of two repetitions. B. TATiΔKu80PIATrx1 (i) or TATiΔKu80PIATrx2-3HA (ii) parasites were grown in ATc as indicated and plastids were counted based on immunofluorescence signal obtained via staining with anti-CPN60 antibody in 100 parasites for each time point (error bars are calculated from 4 independent experiments). Y-axis shows the percentage of parasites containing a plastid. C. qPCR comparing nuclear encoded gene (act1) and apicoplast genome encoded gene (TogoCr29) copy numbers at different time points after down regulation of ATrx1 (i) or ATrx2 (ii). D. qRT-PCR analysis performed with total RNA extracts from TATiΔKu80PIATrx1 (i) or TATiΔKu80PIATrx2-3HA (ii) comparing nuclear encoded mRNA (act1) and apicoplast genome encoded mRNA (TogoCr29) expression levels at different time points after down regulation of ATrx proteins. All the panels show data from parasites grown in the absence of ATc (-ATc) or presence of ATc for 24 (+24h), 48 (+48h) and 72 (+72h) hours. In panel C and D the data was normalized such that copy numbers from each genome from the no ATc treatment sample is 1. All error-bars are SEM. (TIF) [file ppat.1006836.s007.tif]
