# Supplementary material for: Two essential Thioredoxins mediate apicoplast biogenesis, protein import, and gene expression in Toxoplasma gondii
Source: PLoS Pathog. 2018 Feb 22;14(2):e1006836. doi: 10.1371/journal.ppat.1006836 (PMC5823475; doi:10.1371/journal.ppat.1006836)
Supplement: S1 Materials and Methods — (PDF) [file ppat.1006836.s008.pdf]

Supplementary Material and Methods:

***In vitro* insulin turbidity assay**

Insulin aggregation upon reduction of disulphides was monitored by the increase in light scatter at 650 nm. Two µM human thioredoxin or PDI or ATrx2 (expressed and isolated from *E. coli*, as described above) were mixed in 50 mM sodium phosphate pH 7.5, 2 mM EDTA, 0.33 mM DTT and added to a 96 well plate. One hundred µM human insulin (Sigma, I9278) was added to start the reaction and light scatter was measured over time. This experiment was performed once.

Recombinant protein expression and isolation

Expression of 6xHis-tagged ATrx2 from pET28a was carried out at 16 °C for 12 – 15 hours, using 0.1 mM IPTG. Refolding/re-solubilisation was carried out using a 5 mL Histrap column (GE Healthcare), pre-equilibrated with 10 times column volume (CV) of buffer RB (50 mM Na-Phosphate pH = 8, 100 mM NaCl, 8 M Urea, 20 mM Imidazole, 10 mM BME). Sample was filtered and loaded on to the column using a 1 mL/min flow rate. Washing of unbound proteins was done using 10 CV of buffer A (50 mM Na-Phosphate pH=8, 100 mM NaCl, 3 M Urea, 10 % Glycerol, 20 mM Imidazole, 10 mM BME). Elution was done step-wise, first with a gradient of 10 % Buffer B (buffer A lacking urea) for 6 CV, followed by 100 % buffer B for 8 CV. Fraction from peaks at 10 and 100 % buffer B elutions were analysed on SDS-PAGE and then Coomassie-stained.
